# IKBKE activity enhances AR levels in advanced prostate cancer via modulation of the Hippo pathway

**DOI:** 10.1093/nar/gkaa271

**Published:** 2020-04-23

**Authors:** Alex Bainbridge, Scott Walker, Joseph Smith, Kathryn Patterson, Aparna Dutt, Yi Min Ng, Huw D Thomas, Laura Wilson, Benjamin McCullough, Dominic Jones, Arussa Maan, Peter Banks, Stuart R McCracken, Luke Gaughan, Craig N Robson, Kelly Coffey

**Affiliations:** 1 Solid Tumour Target Discovery Laboratory, Translational and Clinical Research Institute, Newcastle University Centre for Cancer, Faculty of Medical Sciences, Newcastle University, Newcastle upon Tyne NE2 4HH, UK; 2 Drug Discovery, Translational and Clinical Research Institute, Newcastle University Centre for Cancer, Faculty of Medical Sciences, Newcastle University, Newcastle upon Tyne NE2 4HH, UK; 3 Bio Screening Facility, Newcastle University, Cookson Building, The Medical School, Framlington Place, Newcastle upon Tyne NE2 4HH, UK

## Abstract

Resistance to androgen receptor (AR) targeting therapeutics in prostate cancer (PC) is a significant clinical problem. Mechanisms by which this is accomplished include *AR* amplification and expression of AR splice variants, demonstrating that AR remains a key therapeutic target in advanced disease. For the first time we show that IKBKE drives AR signalling in advanced PC. Significant inhibition of AR regulated gene expression was observed upon siRNA-mediated IKBKE depletion or pharmacological inhibition due to inhibited *AR* gene expression in multiple cell line models including a LNCaP derivative cell line resistant to the anti-androgen, enzalutamide (LNCaP-EnzR). Phenotypically, this resulted in significant inhibition of proliferation, migration and colony forming ability suggesting that targeting IKBKE could circumvent resistance to AR targeting therapies. Indeed, pharmacological inhibition in the CWR22Rv1 xenograft mouse model reduced tumour size and enhanced survival. Critically, this was validated in patient-derived explants where enzymatic inactivation of IKBKE reduced cell proliferation and AR expression. Mechanistically, we provide evidence that IKBKE regulates AR levels via Hippo pathway inhibition to reduce c-MYC levels at *cis*-regulatory elements within the *AR* gene. Thus, IKBKE is a therapeutic target in advanced PC suggesting repurposing of clinically tested IKBKE inhibitors could be beneficial to castrate resistant PC patients.

## INTRODUCTION

The androgen receptor (AR) is a key molecule in the development and progression of prostate cancer (PC) and as such is a critical therapeutic target. Current androgen-deprivation therapy (ADT) is initially effective at reducing AR signalling and PC progression, but most patients inevitably become resistant to these treatments via multiple mechanisms including *AR* gene amplification and through AR splice variants (1). Therefore, the AR remains a key therapeutic target in ADT-resistant disease and the development of new AR-targeted therapies, although challenging, remains a major unmet clinical need for PC treatment.

AR activity is regulated by numerous post-translational modifications (PTM) which suggests that targeting AR modifying enzymes which enhance AR activity may provide therapeutic benefit when direct AR targeting therapies have failed; particularly as a number of these coregulatory proteins are themselves often dysregulated in PC (2). The best characterized PTM of the AR is phosphorylation (AR-P), where phosphorylation at specific sites determines its biological consequences. For example, phosphorylation at Ser308 by Cyclin D3/CDK11p58 inhibits the transcriptional activity of the AR (3) whilst phosphorylation at Ser81 is linked to transcriptional activation (4). In addition, AR-P can occur under steroid depleted conditions for example, AKT enhances receptor phosphorylation at Ser213 to promote nuclear translocation in response to IGF1 in the absence of androgens (5), and EGF can activate the AR by Ser515 phosphorylation (6). Indeed, many reports have linked the phosphorylation status of the AR with more aggressive disease (7–9). Additionally, many AR co-regulators are similarly regulated via phosphorylation (10,11).

IKBKE (IKKE, IKKi) is a non-canonical I-kappa-B kinase which can be activated by numerous stimuli including TNFα and IL1. It plays a role in numerous signalling pathways, for example it has been shown to phosphorylate CYLD, which in turn activates the NF-κB pathway via deubiquitination of several NF-κB regulator proteins (12). IKBKE can also inactivate the Hippo pathway, which is responsible for regulating organ size, by phosphorylation of LATS1/2 to result in its degradation (13). Furthermore, IKBKE can regulate the stability and nuclear localization of c-MYC in pancreatic ductal carcinoma cell lines (14). In several cancers, IKBKE has been demonstrated to be amplified and overexpressed (12) moreover, it has been found to be oncogenic in breast and ovarian cancer (15,16). Interestingly, in PC, IKBKE exhibits elevated protein expression in cancers compared to normal cells (17).

In this study, we identified IKBKE as a regulator of AR transcriptional activity which engages the Hippo pathway to modulate AR *de novo* synthesis in models of PC. Targeting IKBKE with small molecule inhibitors in both PC cell line xenografts and patient *ex vivo* explant models resulted in reduced tumour volume, inhibition of proliferation and reduced AR expression. Collectively, our data suggest that IKBKE is a viable therapeutic target for the treatment of PC. Interestingly, pharmacological inhibitors of IKBKE are used in treatment of asthma, allergic rhinitis and aphthous ulcers (18,19) and a potential role for these inhibitors has also been identified in obesity related metabolic disorders (20), lung cancer (21) and glioblastoma (13). We propose that IKBKE inhibitors, such as Amlexanox which has been used in clinical trials for Type 2 diabetes (22), may be repurposed to provide therapeutic advantage for advanced PC patients.

## MATERIALS AND METHODS

### Antibodies and constructs

AR (C-19, sc-815, Santa Cruz Biotechnology and clone G122-434, BD), PSA (A0562, Dako), IKBKE (D20G4, Cell Signalling), α-tubulin (clone DM1A, T9026, Sigma), LATS2 (kpm C-2, sc-515579 Santa Cruz Biotechnology), YAP (G-6, sc-376830 Santa Cruz Biotechnology), c-MYC (ab56, Abcam and N262, sc-764, Santa Cruz Biotechnology), TMPRSS2 (H-4, sc-515727, Santa Cruz Biotechnology), PARP1/2 (clone H250, sc-7150, Santa Cruz Biotechnology), FKBP5 (D-4, sc-271547, Santa Cruz Biotechnology), GFP (ab290, AbCam) Ki67 (clone MM1, Novocastra, Leica Biotechnology).

### Compounds

All compounds were purchased in powder form and resuspended in DMSO to a concentration of 10 mM unless otherwise stated. CAY10576 (Santa Cruz Biotechnology), MRT67307 (Stratech Scientific), BX795 (Cambridge BioScience), CYT387 (Adooq Biosciences and Cambridge BioScience), Ruxolitinib (Cambridge BioScience) and R1881 (Sigma) were stored at −80°C for no more than 6 months. Enzalutamide (Selleckchem) was prepared at a concentration of 30 mM and stored at −80°C for no more than 6 months. MG-132 (Sigma) was purchased as a 10 mM stock in DMSO and stored at −80°C.

### Cell culture

VCaP, CWR22Rv1 (23), PC3 and LNCaP cells were obtained from the American Type Culture Collection (Manasses, VA, USA) and LNCaP-7B7 cells (24) were a gift from Professor Jan Trapman (Erasmus Medical Centre, Rotterdam), LAPC4 were a gift from Helmut Klocker (Innsbruck). Cells were maintained in RPMI-1640 media (Sigma) containing 10% (v/v) Foetal Calf Serum (FCS) (Gibco) and 2 mM l-glutamine (Sigma) at 37°C in 5% CO_2_ atmosphere. LNCaP-AI (25) were maintained in RPMI-1640 media containing 10% dextran coated charcoal stripped serum FCS (Biowest) and 2 mM l-glutamine. LNCaP-AI AREIII-LUC cells were generated in house as described in Supplementary Materials and Methods and maintained under the same conditions as LNCaP-AI cells. LNCaP-AR-GFP, LNCaP-EV and LNCaP-YapS127A cells were generated in house as described in Supplementary Materials and Methods. LNCaP-EnzR cells were generated in house by serially maintaining LNCaP cells in RPMI-1640 media containing 10% FCS and 2 mM l-glutamine, supplemented with 10 μM Enzalutamide. LAPC4 cells were maintained in RPMI-1640 media containing 10% FCS and 2 mM l-glutamine (Sigma), supplemented with 1 nM R1881. VCaP cells were maintained in High Glucose DMEM-6171 media (Sigma) containing 10% FCS and 2 mM l-glutamine.

For androgen stimulation assays, cells were grown in RPMI-1640 media (Gibco) supplemented with 10% dextran coated charcoal stripped serum FCS and 2 mM l-glutamine for 72 h, prior to the addition of 1 nM R1881 for indicated lengths of time.

Cell line authentication was performed by short tandem repeat profiling (NewGene, Newcastle upon Tyne, UK) and mycoplasma testing was performed routinely using MycoAlert kit (Lonza, UK).

### siRNA

Cell lines were reverse transfected with individual siRNAs (25 nM) using Lipofectamine RNAiMAX (Invitrogen) according to manufacturer's instructions (for siRNA target sequences see Supplementary Table S1). To assess siRNA knockdown either RNA or protein was collected and subjected to qPCR or western blotting, respectively, as described below.

### Nuclear-cytoplasmic fractionation

LNCaP cells were cultured as previously described and treated with DMSO or IKBKE inhibitors CAY10576 (5 μM) or CYT387 (5 μM) for 16 h. For knockdown experiments, cells were reverse transfected as detailed above and cells collected after 72 h knockdown. Nuclear-cytoplasmic fractionation was performed using the NE-PER Nuclear and Cytoplasmic Extraction Kit (Thermo Scientific) according to the manufacturer's protocol and analysed by western blotting.

### Colony formation assay

LNCaP and LNCaP-Enz cells were reverse transfected with 25 nM N/S or IKBKE siRNA and incubated for 72 h. Cells were then reseeded and incubated for 21 days to allow colony formation. Cells were then fixed and stained with crystal violet. Colonies were counted and the surviving fraction calculated.

### Migration assay

LNCaP cells were either reverse transfected with 25 nM N/S or IKBKE siRNA and incubated for 72 h or pre-treated with CAY10576 for 24 h prior to seeding into migration chambers in basal media and inserted into wells containing serum supplemented media as a chemoattractant. Cells were incubated for 16 h, then inner chambers scrubbed to remove cells which did not migrate through the pores of the membrane. Cells which had migrated to the other side of the membrane were then fixed in ice cold methanol and stained with haematoxylin. Cells were then imaged using Aperio CS2 Digital Pathology scanner (Leica) and counted.

### Western blotting

Western analysis was performed as previously described (26).

### Quantitative polymerase chain reaction

RNA was isolated using TRIzol (Invitrogen) according to the manufacturer's protocol. RNA was quantified using Nanodrop spectrophotometer, and 200 ng of RNA was reverse transcribed using 1× Moloney Murine Leukemia Virus (MMLV) buffer (Promega), 60 U MMLV reverse transcriptase, 0.5 μM oligo d(T)_15_ (Promega) and 0.4 mM dNTPs (Bioline) per reaction. Quantitative reverse transcriptase–polymerase chain reaction (qPCR) was performed in triplicate for genes of interest using Platinum® SYBR^®^ green qPCR SuperMix-UDG with ROX (Invitrogen) in 384-well clear optical reaction plates using the QuantStudio 7 Flex real-time PCR system (Applied Biosystems) according to the manufacturer's protocol. Data were normalized against the house keeping gene *hypoxanthine phosphoribosyltransferase 1* (*hprt1*). Primers sequences are described in Supplementary Table S2.

### Chromatin immunoprecipitation

Chromatin immunoprecipitation (ChIP) assays were performed in LNCaP and LNCaP-AR-GFP cells reverse transfected with either 25 nM N/S or IKBKE siRNA for 72 h, according to the protocol described by Schmidt *et al.* (27). Briefly, 100 μg of DNA and 2 μg of AR antibody (Santa Cruz Biotechnology C-19), c-MYC antibody (ab55, AbCam) or GFP antibody (ab290, AbCam) and equal quantities of non-specific isotype control antibodies (DAKO) were used. Using primers specific to AREIII and AREII PSA enhancer element, AREI PSA promoter region and c-MYC binding site within the AR promoter (Supplementary Table S3) purified DNA fragments were analysed by qPCR. Data were calculated as % input and presented as the average fold difference of % input between different experimental arms for three independent experiments.

### Cell growth analysis

Cell growth was assessed using the IncuCyte ZOOM live cell imager (Essen) according to the manufacturer's recommendations. In each case, cells were treated and grown in their respective normal growth media, unless otherwise stated. For knockdown experiments, cells were reverse transfected with 25 nM siRNA using RNAiMax according to the manufacturer's protocol. For compound assays, cells were seeded and allowed to adhere for 24 h, prior to the addition of compound. Images were taken every 6 h for at least three doubling times. The IncuCyte ZOOM 2015A software package was trained to identify cells from each cell line and measure the % confluence of each well. Cell confluence was normalized for each well at the 0 time point and the relative change in cell confluence was calculated for each time point thereafter. Data were presented as the mean relative cell confluence at each time point from at least three independent experiments.

### Cell cycle analysis

LNCaP cells were reverse transfected with 25 nM siRNA using RNAiMax according to the manufacturer's protocol and grown for 96 h. Cells were collected in 100 μl citrate buffer (0.25 M sucrose, 40 mM sodium citrate, pH 7.6). DNA staining and lysis buffer (20 μg/ml propidium iodide (Sigma), 0.5% NP-40 (Calbiochem), 250 μg/ml RNaseA (Qiagen) 0.5 mM ethylenediaminetetraacetic acid (EDTA), in PBS) was prepared and 400 μl added to the cell suspension and incubated for 40 mins in the dark at 4°C. Using FACScan together with CellQuest software (Beckton Dickenson) 10 000 events were collected and analysed using Cyflogic software (CyFlo Ltd). The percentage of cells in each cell cycle phase was determined and data presented as the mean % of cells from three independent experiments +/− SEM. Student's t-test was used to determine statistical significance between the experimental conditions.

### Animal studies

CWR22Rv1 cells (1 × 10^6^ per mouse) were implanted subcutaneously in 0.2 ml Matrigel into CD1 nude mice (Charles Rivers, Kent, UK). Once palpable tumours were detected at an average volume of 100 mm^3^, CYT387 was administered via daily oral gavage at a dose of 100 mg/kg or vehicle control. Tumour volume was measured using callipers according to the equation *a*^2^ × *b*/2, (*a* = smallest measurement; *b* = largest measurement). Median relative tumour volume (RTV) was calculated, where RTV1 = tumour volume on day 1 of treatment for each mouse. Time to RTV4, defined as the time it takes for tumours to reach four times the initial volume, was calculated as described previously (28). Murine body weight was also measured following daily dosing.

The *in vivo* experiments were approved by the local animal welfare ethics review body (AWERB) and performed according to ethical guidelines set out by Workman *et al.* under a UK home office licence in accordance with the Animals (Scientific Procedures) Act 1986 (29).

### Explant culture of human prostate tumours

Following ethical approval, human PC tissue was obtained with written, informed consent from patients with known PC undergoing robotic radical prostatectomy (RRP) or channel transurethral resection of the prostate (chTURP).

*Ex vivo* explant cultures were established as described by Centenera *et al.* (30). Collected tumour tissue was dissected into ∼1–2 mm^3^ pieces and placed on pre-soaked 1 cm^3^ gelatin sponges (Spongostan Dental) in a 24-well plate containing 500 μl RPMI-1640 HEPES modification medium (Sigma-Aldrich) with 10% foetal calf serum and 1% L-Glutamine supplemented with 1× antibiotic anti-mycotic solution (Sigma-Aldrich), 0.01 mg/ml hydrocortisone (Sigma-Aldrich), 0.01 mg/ml bovine insulin solution (Sigma-Aldrich) and synthetic androgen 1 nM R1881. Treated explants were cultured in *ex vivo* medium containing CYT387 (10 μM). Control explants were treated with a matched volume of DMSO. Explants were cultured at 37°C with 5% CO_2_ for 48 or 72 h before being formalin-fixed and paraffin embedded. Patient information detailed in Supplementary Table S4.

### Immunohistochemistry

Four μm-thick sections of formalin-fixed paraffin-embedded (FFPE) explant tissue were stained for the proliferative marker Ki67. Automated immunohistochemistry (IHC) was carried out on a Discovery XT staining module (Roche). Antigen was retrieved by incubating slides in Cell Conditioning Solution (Roche). Endogenous peroxidase activity was reduced by incubating slides in Inhibitor CM (Roche). Slides were incubated in Ki67 primary antibody (Novocastra; 1:100) or Anit-AR primary antibody (clone G122-434, BD) for 1 h at 37°C followed by HRP-conjugated Discovery OmniMaP HRP secondary antibody (Roche) for 30 min. A ChromoMap DAB kit (Roche) was used for detection. Slides were counterstained in hematoxylin and mounted in DPX. Stained slides were imaged using an Aperio CS2 Digital Pathology scanner (Leica). Regions of interest (i.e. epithelial cells) were manually annotated and the percentage of Ki67 positive cells was determined using Aperio Image Analysis software (Leica) in a minimum of four representative 40× magnification regions containing a total of at least 1000 nuclei. Positive control tissue for Ki67 is shown in Supplementary Figure S8 alongside no primary control staining. Serial sections were also stained for p63 (basal cell marker identifying benign regions) and AMACR (cancer cell marker) to define regions of cancer (Supplementary Figure S9).

### Statistical analysis

The numbers of independent experimental repeats are detailed within figure legends. Where data is expressed as mean ± SEM of three or more independent experiments, statistical analysis was performed to indicate significance. Specifically, Student's *t*-test was used for data sets containing two experimental arms and one-way ANOVA with Greenhouse-Geisser correction and Dunnett's multiple comparison test was performed where there were three or more experimental arms to the experiment. Two-way ANOVA with Dunnett's multiple comparison test was performed on data with two variables. Tests were undertaken in GraphPad Prism 6 software.

### Study approval

The NHS Health Research Authority NRES Committee North East, Newcastle and North Tyneside, has provided approval for human tissue studies in the laboratory of Dr. McCracken (REC reference: 15/NE/0400).

The *in vivo* experiments were approved by the local animal welfare ethics review body (AWERB) and the Home Office in accordance with the Animals (Scientific Procedures) Act 1989.

## RESULTS

### IKBKE is required for AR transcriptional activity

**Figure 1.**
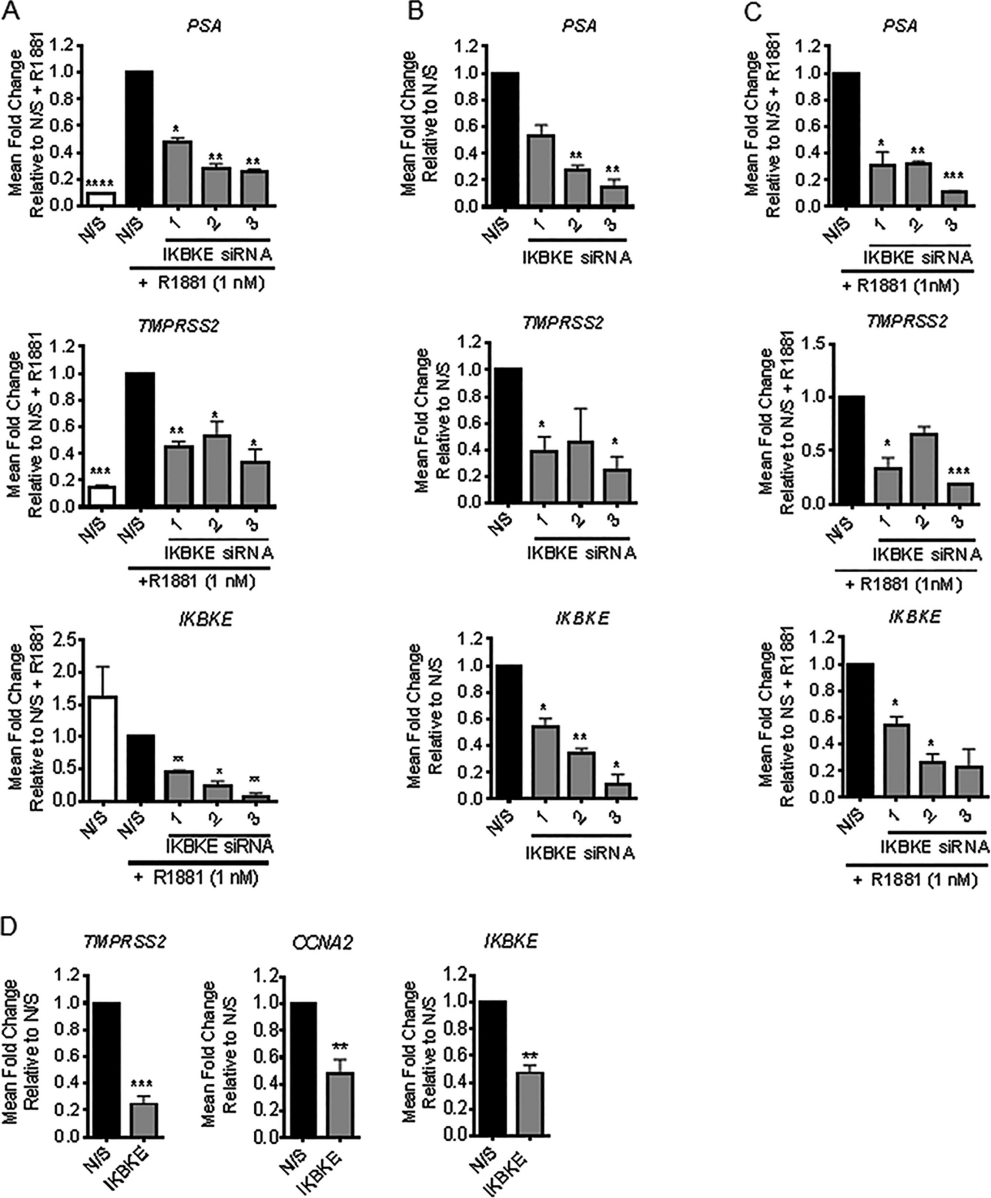
IKBKE is required for AR signalling. (**A**) LNCaP cells were reverse transfected in steroid depleted media (SDM) with either N/S or three individual siRNAs against IKBKE. After 72 h, cells were stimulated with R1881 (1 nM) then *PSA, TMPRSS2* and *IKBKE* mRNA expression determined at 24 h by qPCR (n = 3). (**B**) LNCaP cells were reverse transfected in full media with either N/S or three individual siRNAs against *IKBKE*. After 72 h, *PSA, TMPRSS2* and *IKBKE* mRNA expression determined by qPCR (*n* = 3). (**C**) LNCaP-AI were transfected in SDM with either N/S or three pooled siRNAs against IKBKE. After 72 h, cells were stimulated with R1881 (1 nM) then *PSA, TMPRSS2* and *IKBKE* mRNA expression determined at 24 h by qPCR (*n* = 3). (**D**) CWR22Rv1 cells were transfected with either N/S or pooled siRNAs against IKBKE in SDM, 72 h later cells were lysed and assessed for *TMPRSS2, CCNA2* and *IKBKE* expression by qPCR (*n* = 3). qPCR data is expressed as mean fold change of three experimental repeats ± SEM. One-way ANOVA paired Dunnetts multiple comparison test * *P* < 0.05, ** *P* < 0.01; *** *P* < 0.001 **** *P* < 0.0001.

To identify protein kinases required for AR mediated transcriptional regulation, a primary unbiased siRNA library screen (Dharmacon) was performed, in which each kinase in the human genome was individually depleted in androgen sensitive LNCaP-7B7 (24) cells in the presence of the synthetic AR agonist, R1881. This LNCaP derivative cell line was selected to enable high-throughput assessment of AR transcriptional activity by measuring AR regulated luciferase activity as a surrogate. Candidate validation was performed by a secondary screen, consisting of independent pools of MISSION (Sigma) siRNAs, identifying a role for the threonine serine kinase, IKBKE in AR transcriptional activation (Supplementary Figure S1A). Validation using a deconvoluted siRNA pool in parental LNCaP cells confirmed that IKBKE knockdown significantly reduced expression of the AR target genes, prostate specific antigen (*PSA)* and transmembrane serine protease 2 (*TMPRSS2)* in the presence or absence of exogenous androgen (R1881) stimulation (Figure 1A, B). Additionally, parallel screening performed in an androgen–independent version of the LNCaP cell line, LNCaP-AI AREIII-LUC, also confirmed IKBKE as a regulator of AR transcriptional activity both in the presence and absence of androgenic stimulation (Supplementary Figure S1B, C). Consistent with this result, in the parental LNCaP-AI cells IKBKE knockdown diminished *PSA* and *TMPRSS2* levels (Figure 1C). Due to the role of constitutively active AR splice variants (AR-Vs) in the development of advanced PC we predicted that IKBKE would influence AR-V regulated gene expression. Variant expressing CWR22Rv1 cells were examined for *TMPRSS2* and *CCNA2* expression subsequent to IKBKE knockdown in steroid depleted media where only AR-Vs, which lack the ligand binding domain, will be active. Indeed, constitutively active AR-V mediated transcriptional activation of *TMPRSS2* and *CCNA2* was impeded by IKBKE knockdown (Figure 1D). Taken together these results validate a critical role for IKBKE signalling in maintaining AR activity throughout PC progression.

### IKBKE regulates AR mRNA levels

**Figure 2.**
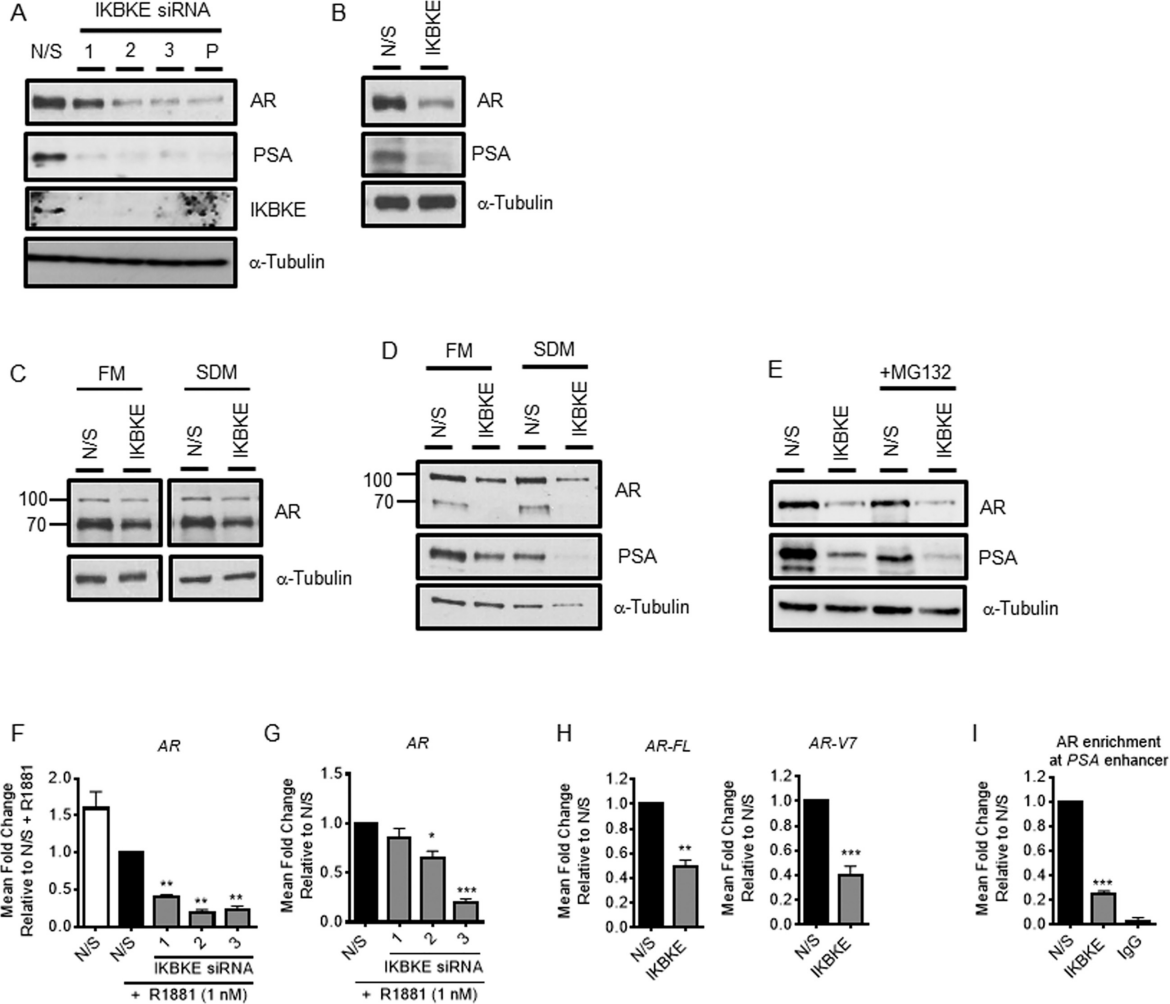
IKBKE regulates AR mRNA expression. (**A**) LNCaP cells were transfected in serum containing media (FM) with N/S or three individual/pooled siRNAs against IKBKE. After 72 h, (A) PSA, AR and IKBKE expression was determined by immunoblotting. (**B**) LNCaP-AI cells were transfected in steroid depleted media (SDM) with N/S or three pooled IKBKE siRNAs. After 72 h, PSA and AR expression was assessed by immunoblotting. (**C**) CWR22Rv1 and (**D**) VCaP cells were transfected with N/S or pooled IKBKE siRNAs and grown in either FM or SDM as indicated. After 72 h, PSA and AR expression were determined by immunoblotting. (**E**) LNCaP cells were transfected with N/S or pooled IKBKE siRNAs. After 40 h, the proteasomal inhibitor MG132 (20 μM) was applied for 16 h. PSA and AR expression was determined by immunoblotting. (**F**) LNCaP cells were transfected in SDM with N/S or three individual IKBKE siRNAs. After 72 h, R1881 was applied for 24 h then *AR* mRNA quantified by qPCR (*n* = 3). One-way ANOVA paired Dunnetts multiple comparison test. (**G**) LNCaP-AI cells were transfected in SDM with N/S or three individual IKBKE siRNAs. After 72 h, cells were stimulated with R1881 (1 nM) then *AR* mRNA expression determined at 24 h by qPCR (*n* = 3). One-way ANOVA paired Dunnetts multiple comparison test (**H**) CWR22Rv1 cells were transfected with N/S or IKBKE siRNA pool in SDM. After 72 h full length *AR* (*AR-FL*) and *AR-V7* gene expression was analysed by qPCR (*n* = 3). Student's *t*-test. (**I**) LNCaP cells were transfected with N/S or pooled IKBKE siRNAs. After 72 h, chromatin immunoprecipitation for AR was performed and recruitment to the PSA enhancer assessed by qPCR (*n* = 3). One-way ANOVA paired Dunnetts multiple comparison test * *P* < 0.05, ** *P* < 0.01; *** *P* < 0.001. P: pooled siRNA.

As IKBKE modulates the expression of genes which can be driven by both full length AR and AR-Vs we questioned whether IKBKE could alter AR levels as a mechanism underlying these observations. Firstly, we examined the effects of IKBKE knockdown using both a pool of three siRNAs and individual siRNAs in LNCaP cells growing in serum containing media. After 72 h, western blotting confirmed IKBKE knockdown, and PSA expression was inhibited but further demonstrated that total AR protein levels were found to be robustly reduced (Figure 2A). LNCaP-AI cells, a model of androgen independent PC, grown in steroid depleted media similarly exhibited reduced AR protein levels upon IKBKE knockdown (Figure 2B, Supplementary Figure S2A). In addition, to ensure these effects were not cell line dependent, the effect of IKBKE knockdown was extended to the androgen dependent LAPC4 cell line (Supplementary Figure S2B) and AR-V expressing models of castrate resistant prostate cancer (CRPC), CWR22Rv1 and VCaP cell lines, where consistently it was observed that both under androgen deprived (SDM) and androgen replete (FM) conditions, both AR and AR-V expression was reduced (Figure 2C, D; Supplementary Figure S2C). Combined, this data suggests that IKBKE plays a robust role in the regulation of AR levels within multiple pre-clinical cell line models, representative of androgen sensitive and CRPC.

Two alternative mechanisms may explain how IKBKE could modulate AR protein levels; either through proteasomal-mediated regulation of AR protein turnover or by regulation of AR mRNA. To determine whether AR down regulation is a consequence of enhanced AR turnover in response to IKBKE knockdown we monitored AR expression upon IKBKE knockdown in the presence of the proteasomal inhibitor, MG132. Interestingly, we observed that AR expression could not be restored by the addition of MG132 suggesting that IKBKE does not play a role in AR destruction by the proteasome (Figure 2E). Secondly, the role of IKBKE in the regulation of AR mRNA was tested by qPCR following IKBKE knockdown in LNCaP and LNCaP-AI cells. This revealed that IKBKE knockdown (Figure 1A, B) caused a significant reduction in AR mRNA levels in LNCaP (Figure 2F) and LNCaP-AI (Figure 2G) cells. Furthermore, both full length and AR-V7 mRNA transcripts were significantly reduced in CWR22Rv1 cells upon IKBKE knockdown (Figures 1D and 2H). As we have observed a robust reduction in both AR mRNA and protein levels in response to IKBKE knockdown we asked whether this consequently led to reduced AR association with androgen responsive elements in promoter-enhancer regions of AR regulated genes, causing reduced target gene expression. ChIP in LNCaP cells depleted of IKBKE revealed a significant ∼75% reduction in AR binding to the *PSA* enhancer (*P* < 0.001) (Figure 2I). Together, this data suggests that IKBKE plays a role in the regulation of AR mRNA which reduces the levels of AR protein available to positively associate with AR regulated genes as its primary mechanism of action on the AR signalling pathway.

### IKBKE can affect AR transcriptional activity independent of its effects on AR mRNA

**Figure 3.**
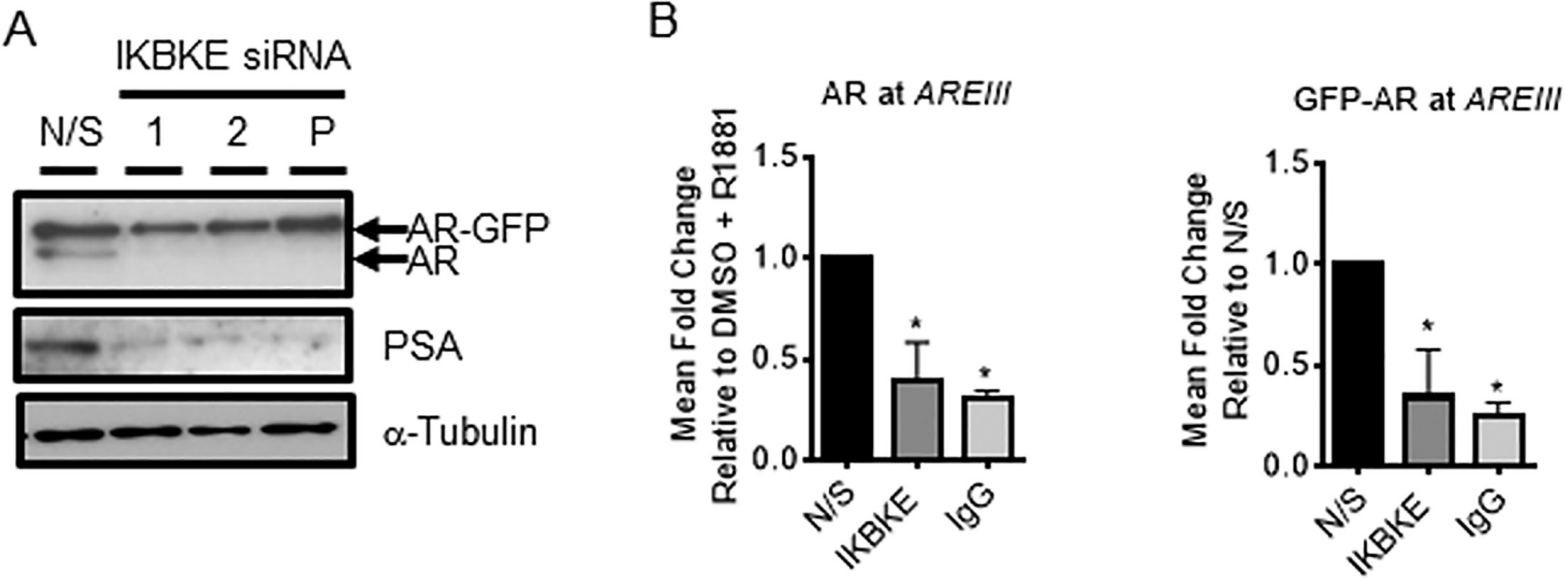
IKBKE inhibits AR transcriptional activity. LNCaP-AR-GFP cells were reverse transfected with individual IKBKE targeting siRNAs or a pool of siRNA 1 and 2 for 72 h. (**A**) Lysates were then collected and western blotting performed for PSA, endogenous AR and GFP tagged AR. Alpha tubulin was used as a loading control. Blots are representative of 2 experimental repeats. (**B**) Chromatin immunoprecipitation using either anti-AR antibody, anti-GFP antibody or IgG control was performed and recruitment to the PSA enhancer was assessed by qPCR (*n* = 3). Data is expressed as mean fold change relative to N/S control ± SEM. Unpaired one-way ANOVA * *P* < 0.05.

Due to the severity of the effect of IKBKE knockdown on AR protein levels as a consequence of inhibited AR mRNA expression it is challenging to determine whether IKBKE can also modulate AR transcriptional activity independently of the effects on AR levels. To address this question we utilized a LNCaP cell line which has been engineered to constitutively express AR-GFP from a constitutive CMV promoter (Supplementary Materials and Methods; Supplementary Figure S3A, B). Firstly, knockdown of IKBKE was performed and PSA expression investigated by western blotting after 72 h. This revealed that IKBKE can also modulate the transcriptional activity of the AR independent of its effect on endogenous AR mRNA expression with a robust reduction in PSA protein levels being observed with IKBKE knockdown even though AR-GFP expression was unaffected (Figure 3A; Supplementary Figure S3C). Finally, to confirm that AR-GFP can bind to the PSA enhancer we performed ChIP assays under these same conditions. Upon IKBKE knockdown we observed a significant 61.3% reduction in AR association (*P* < 0.05) with the PSA promoter-enhancer region comparable with control IgG background levels (Figure 3B; Supplementary Figure S3D). Furthermore, ChIP using an anti-GFP antibody confirmed that AR chromatin association is affected independent of transcriptional down regulation of endogenous AR levels, with a significant 65.6% reduction similar to IgG control background levels (Figure 3B; Supplementary Figure S3E).

Collectively, these data indicate that IKBKE knockdown mediates its effects on AR signalling mainly by suppressing AR mRNA expression but also by inhibiting protein activity suggesting that IKBKE is a major node of AR regulation in PC cells.

### Pharmacological antagonism of IKBKE suppresses AR expression and transcriptional activity

To determine whether IKBKE is clinically relevant in PC we investigated publically available data in cBioportal in the first instance. Indeed, upon investigation of all prostate data sets, it appears that IKBKE is amplified in CRPC (27.14%; 19/70 cases) compared to prostate adenocarcinoma (1.74%; 82/4712 cases) and normal prostate (0%; 0/313 cases) (Supplementary Figure S4A). Furthermore, genetic alterations in IKBKE within these data sets is significantly associated with poorer survival (Supplementary Figure S4B) suggesting that therapeutic targeting of IKBKE may provide patient benefit.

**Figure 4.**
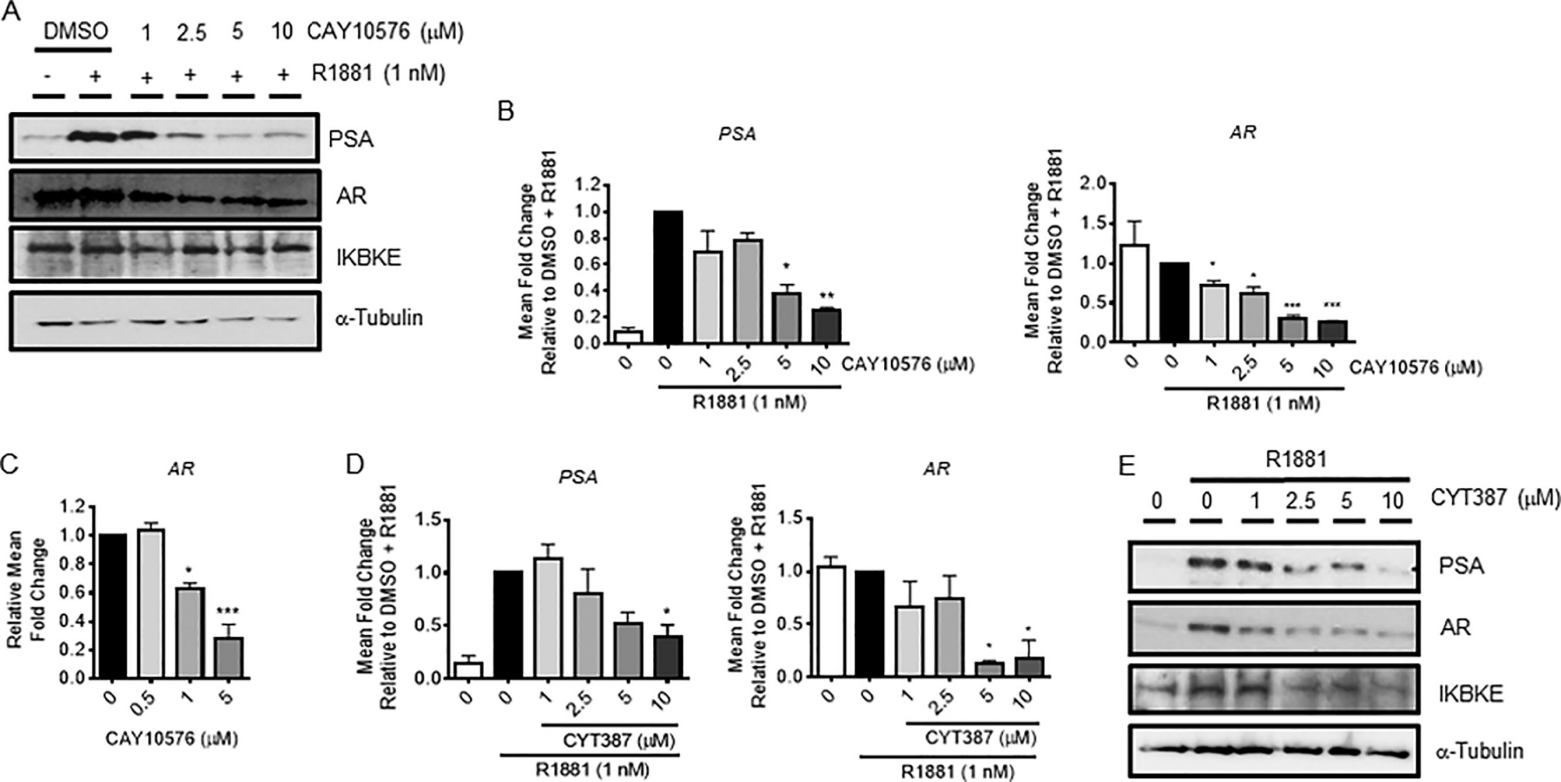
IKBKE catalytic activity is required for AR protein expression and transcriptional activation. After 72 h steroid depletion, LNCaP cells were pre-treated with either vehicle (DMSO) or increasing concentrations of IKBKE antagonist, CAY10576, for 8 h followed by treatment with either vehicle (DMSO) or 1 nM R1881 for a further 24 h. (**A**) Lysates were assessed for PSA, AR and IKBKE protein expression by immunoblotting or resultant cDNA was assessed for (**B**) *PSA* mRNA and *AR* mRNA expression by qPCR (n = 2). (**C**) LNCaP cells grown in full media were treated with either vehicle or increasing concentrations of CAY10576 for 16 h. *AR* mRNA levels were determined by qPCR (*n* = 2). (**D**) After 72 h steroid depletion, LNCaP cells were pre-treated with either vehicle or increasing concentrations of CYT387 for 8 h followed by treatment with either vehicle or 1 nM R1881 for a further 24 h. *PSA* and *AR* mRNA expression determined by qPCR (*n* = 3) and (**E**) PSA, AR and IKBKE protein expression was determined by immunoblotting. Data is expressed as mean fold change of independent experiments ± SEM. Western blots are representative of at least 2 experimental repeats. α-tubulin was used as a loading control. One-way ANOVA, Dunnett's multiple comparisons test * *P* < 0.05, ** *P* < 0.01, *** *P* < 0.001.

IKBKE inhibitors have been developed and used in clinical trials to treat patients with Type 2 diabetes (22). Therefore, if the kinase activity is responsible for effects on AR levels these available inhibitors may expedite new PC therapies. To discriminate between the kinase-dependent and -independent functions of IKBKE in AR signalling, LNCaP cells were treated with the small molecule IKBKE kinase inhibitor, CAY10576 (31). Consistent with the observations following IKBKE depletion, pharmacologically antagonizing IKBKE either prior to R1881 stimulation in steroid depleted media led to a robust, dose-dependent reduction in PSA and AR protein expression (Figure 4A), as well as *PSA* and *AR* mRNA levels (Figure 4B) at effective concentrations previously described (20). Similarly, a significant dose dependent decrease in AR mRNA levels was seen when cells were treated in serum containing media (Figure 4C). To confirm these findings, additional IKBKE inhibitors were evaluated. Firstly, BX795, which can also inhibit TBK1, PDK1, p38 and JNK resulted in a significant reduction in *PSA* mRNA and protein levels and also reduced AR levels consistent with the CAY10576 data (Supplementary Figure S5A, B). A second generation version of BX795, termed MRT67307, which does not inhibit JNK or p38, demonstrated consistent inhibitory effects on PSA levels (Supplementary Figure S5C, D). Thirdly, CYT387, which was developed as a JAK inhibitor but can also target IKBKE and TBK1, resulted in a significant dose dependent reduction in *PSA* and *AR* mRNA and protein levels in LNCaP cells (Figure 4D, E). Furthermore, total IKBKE protein levels appear to be unaffected in response to IKBKE inhibition with the exception of CYT387, consistent with other published data. Taken together, these data support a role for IKBKE catalytic activity in AR mediated transcription by facilitating *de novo* AR synthesis.

### IKBKE regulates AR mRNA expression via the Hippo pathway

As IKBKE is a cytosolic protein which can influence AR mRNA production and AR activity we hypothesize that other proteins are involved as intermediaries between IKBKE and AR. Firstly, we suspected NF-κB signalling may be implicated as IKBKE has previously been shown to stimulate NF-κB activation via phosphorylation at S468 to enhance RelA nuclear localization (32) as well as regulate AR mRNA expression (33). However, we did not observe any p65 in the nucleus of LNCaP cells under non-silencing (N/S) conditions (Supplementary Figure S5E) suggesting that NF-κB is not activated and therefore unlikely to be playing a role in the regulation of AR mRNA in these experiments.

IKBKE is also a regulator of the Hippo pathway whereby it both directly and indirectly, via large tumour suppressor serine/threonine kinases 1/2 (LATS1/2), controls the stability and nuclear translocation of the transcriptional co-activator Yes-associated protein (YAP) (13). The Hippo pathway has been found to be frequently deregulated in cancer where it plays a critical role in the regulation of cell fate and tissue growth. Interestingly, YAP has recently been identified as a direct co-activator of the AR (34) and may indirectly regulate AR mRNA via the transcriptional regulation of its downstream target gene, *c-MYC* which has been shown to modulate AR mRNA production (34,35). Therefore, we hypothesized that YAP and c-MYC are key modulators of IKBKE mediated effects on AR signalling. To test this theory, the effects of both IKBKE depletion and pharmacological antagonism on YAP and c-MYC were investigated.

**Figure 5.**
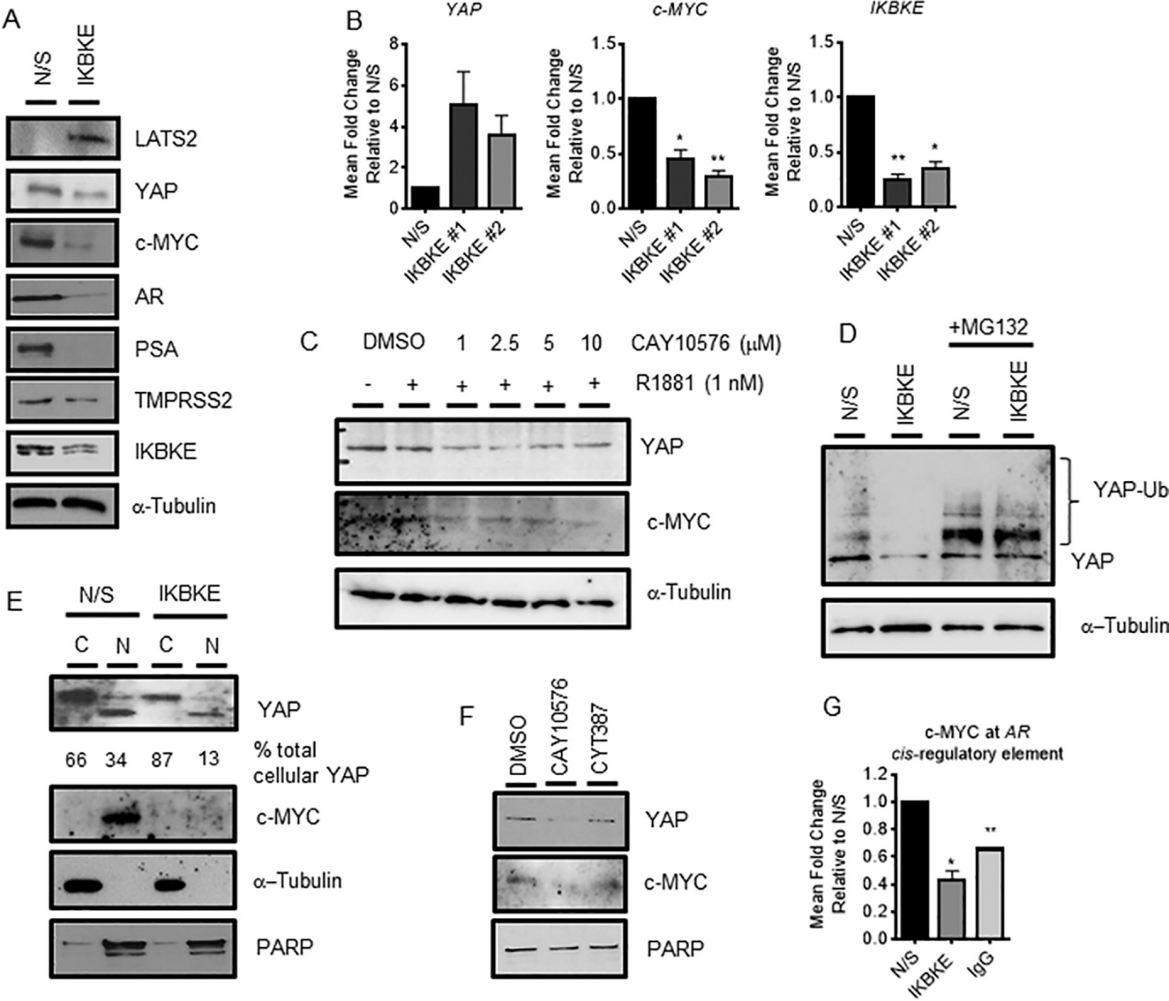
IKBKE influences AR mRNA expression via the Hippo pathway. LNCaP cells were reverse transfected in full media (FM) with either N/S or three pooled siRNAs against *IKBKE*. After 72 h, protein expression was assessed by (**A**) immunoblotting and (**B**) *YAP*, *c-MYC* and *IKBKE* mRNA expression determined by qPCR (*n* = 3). (**C**) After 72 h steroid depletion, LNCaP cells were pre-treated with vehicle (DMSO) or increasing concentrations of IKBKE antagonist, CAY10576, for 8 h followed by treatment with vehicle or 1 nM R1881 for a further 24 h. YAP and c-MYC protein levels were assessed by immunoblotting. (**D**) LNCaP cells were reverse transfected in FM with either N/S or pooled siRNAs against *IKBKE*. After 72 h, MG132 (20 μM) was applied and incubated for a further 16 h. YAP levels were determined by immunoblotting. (**E**) LNCaP cells were reverse transfected in FM with either N/S or pooled siRNAs against *IKBKE*. After 72 h, nuclear-cytoplasmic fractionation was performed with YAP and c-MYC localization determined by immunoblotting. PARP was used as a nuclear loading control and α-tubulin as a cytoplasmic loading control. Densitometry was performed on YAP expression and normalized to loading controls using ImageJ software. Data is expressed as a % of total cellular YAP. (**F**) LNCaP cells were treated with either vehicle or CAY10576 (5 μM) or CYT387 (5 μM) for 16 h prior to nuclear-cytoplasmic fractionation. Nuclear YAP and c-MYC levels were determined by immunoblotting. (**G**) LNCaP cells were reverse transfected in FM with either N/S or pooled siRNAs against *IKBKE* and grown for 48 h prior to ChIP analysis using antibodies specific to c-MYC and isotype controls (IgG). Recruitment to the c-MYC binding site within the AR gene was assessed by qPCR. Data are an average of 3 independent experiments ± SEM. One-way ANOVA paired Dunnetts multiple comparison test * *P* < 0.05, ** *P* < 0.01.

SiRNA-mediated depletion of IKBKE in LNCaP cells grown in serum-containing media resulted in a robust reduction in YAP and c-MYC protein expression, alongside a reduction in AR protein expression and AR target genes, PSA and TMPRSS2 (Figure 5A). However *YAP* mRNA levels increased 5-fold (±SEM 1.64) and 4 fold (±SEM 0.96) with two independent siRNA sequences, whilst *c-MYC* mRNA was significantly reduced by >50% in response to significant IKBKE knockdown (Figure 5B) suggesting that YAP is down-regulated post-translationally by IKBKE consistent with other reports in the literature (36). Moreover, inhibition of IKBKE with CAY10576 led to a reduction in both YAP and c-MYC protein levels (Figure 5C). Interestingly, we demonstrated that depletion of IKBKE led to a dramatic increase in LATS2 expression (Figure 5A). As LATS2 is the kinase responsible for inactivating YAP by promoting nuclear exclusion and degradation this further strengthens the hypothesis that YAP post-translational regulation is involved in IKBKE mediated effects. Indeed, upon proteasomal inhibition with MG132, the levels of YAP protein following IKBKE knockdown were rescued (Figure 5D).

Upon investigation into the cellular localization of YAP in LNCaP cells in response to IKBKE knockdown or pharmacological inhibition, a decrease in nuclear YAP was observed alongside a robust reduction in nuclear c-MYC (Figure 5E, F). As previously mentioned, c-MYC can regulate and promote AR *de novo* mRNA synthesis by binding directly to the *AR* gene itself (35), therefore to test whether the reduction in nuclear c-MYC levels results in a decrease in c-MYC binding to the AR gene, we performed IKBKE knockdown followed by ChIP. As expected, upon IKBKE knockdown in LNCaP cells the presence of c-MYC at the AR gene was significantly decreased by 60% (Figure 5G). Taken together, these findings suggest that via the regulation of Hippo signalling, in particular the stability and cellular compartmentalization of the transcriptional co-activator YAP and therefore *de novo* synthesis of its target gene c-MYC, IKBKE influences *AR* mRNA expression to significantly antagonize AR mediated transcriptional activity.

### Expression of constitutively active Yap rescues AR mRNA expression

**Figure 6.**
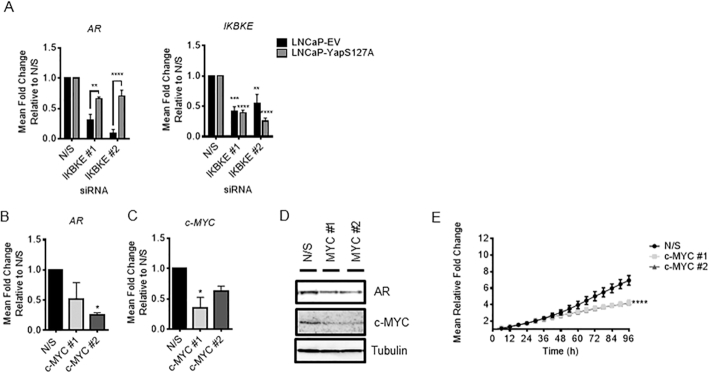
IKBKE effects on AR expression are phenocopied by c-MYC knockdown and partially rescued by overexpression of constitutively active Yap. (**A**) LNCaP-EV and LNCaP-Yap-S127A were reverse transfected with two individual siRNA sequences targeting IKBKE. RNA was collected after 72 h and assessed for expression of AR and IKBKE by qPCR (*n* = 3). (**B**) LNCaP cells were reverse transfected with either N/S or two independent siRNA sequences targeting c-MYC. After 72 hours, RNA was collected and AR mRNA and (**C**) c-MYC expression levels determined by QPCR (*n* = 3). One-way ANOVA, Dunnett's multiple comparisons test * *P* < 0.05, ** *P* < 0.01, *** *P* < 0.001, **** *P* < 0.0001. (**D**) Concurrently, protein lysates were also collected and tested for AR and c-MYC expression by western blotting. (**E**) LNCaP cells were reverse transfected in full media with either N/S or two independent siRNA sequences targeting c-MYC and cell confluency assessed using the IncuCyte Zoom Live Cell Imager every 6 h for 96 h (*n* = 3). 2way ANOVA, Dunnett's multiple comparisons test **** *P* < 0.0001.

To confirm that Yap and c-MYC are mediating AR regulation in response to IKBKE knockdown we generated an LNCaP cell line that overexpresses a constitutively active Yap (Supplementary Figures S10–S11). As this version of Yap, which is mutated at Serine 127, cannot be phosphorylated it remains in the nucleus where it can activate gene transcription. IKBKE was knocked down in these cells and their empty vector control cell line and AR expression determined by qPCR after 72 hours. In order to compare results between these two cell lines we ensured that the level of IKBKE knockdown was as similar as possible and looked at fold changes in AR mRNA levels. We observed that upon IKBKE knockdown, expression of IKBKE in LNCaP-EV was similar to that observed in the parental cell line shown in Figure 2, particularly for siRNA IKBKE #2. When compared to the LNCaP-Yap S127A cell line we see a significant rescue in AR mRNA expression to around a 40% reduction with both siRNA sequences compared to 60–90% reduction in the LNCaP-EV cell line when a 60% knockdown is achieved (Figure 6A).

In addition, to further confirm our proposed mechanism of IKBKE action we investigated the effects of c-MYC knockdown on AR mRNA levels. In support of our rescue experiment data we found that upon knockdown of c-MYC, a reduction in AR mRNA and protein levels in LNCaP cells (Figure 6B–D) as well as a significant reduction in LNCaP proliferation is observed (Figure 6E). Taken together these two lines of evidence support our proposed mechanism that IKBKE functions via Yap and c-MYC to impact AR mRNA expression.

### IKBKE is required for PCa cell growth and progression.

**Figure 7.**
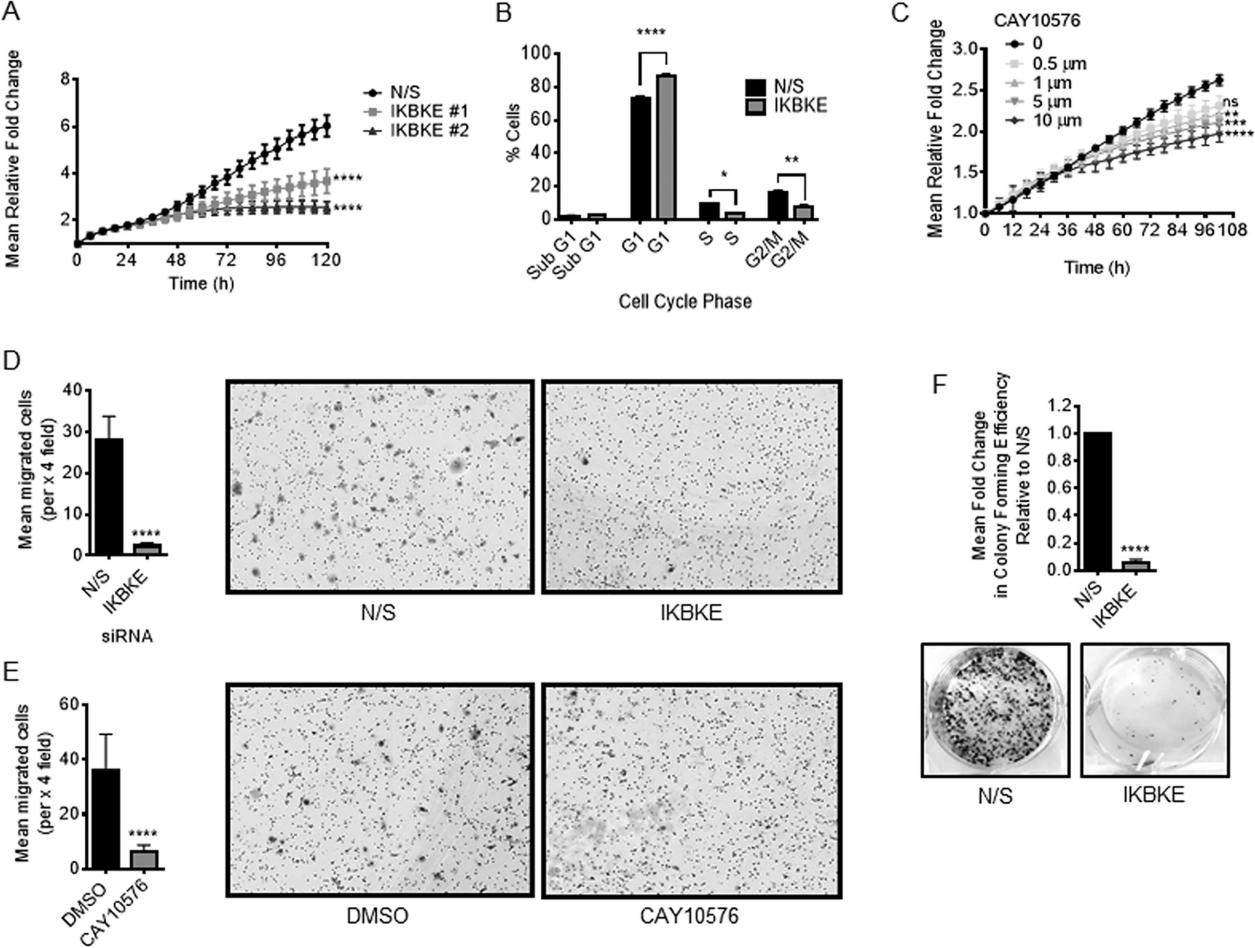
IKBKE is required for PC cell growth and survival. (**A**) LNCaP cells were reverse transfected in full media with either N/S or pooled siRNAs against IKBKE and cell confluency assessed using the IncuCyte Zoom Live Cell Imager every 6 h for 96 h (*n* = 4). Two-way ANOVA, Dunnett's multiple comparisons test **** *P* < 0.0001. (**B**) LNCaP cells were treated as in (A) prior to harvesting at 96 h for cell cycle analysis by propidium iodide flow cytometry. Data shows the % of cells in each phase of the cell cycle (*n* = 3). Pearson's Chi-squared test revealed a significant difference between N/S and IKBKE knockdown treatments (*P* < 10^−15^); two-way ANOVA (variables: cell cycle phase and siRNA treatment) with multiple comparisons reveals significant differences between individual phases. * *P* < 0.05, ** *P* < 0.01, **** *P* < 0.0001. (**C**) LNCaP cells were treated with either vehicle or increasing concentrations of the IKBKE inhibitor CAY10576 in full media and cell confluency assessed as described as in A (*n* = 3). Two-way ANOVA, Dunnett's multiple comparisons test, ns – not significant, ** *P* < 0.01, *** *P* < 0.001, **** *P* < 0.0001. (**D**) LNCaP cells were reverse transfected in full media with either N/S or pooled siRNAs against IKBKE and incubated for 72 h prior to cell counting and equal numbers being resuspended in basal media and dispensed into Boyden chambers suspended in full media as a chemoattractant, in triplicate. (**E**) Similarly, LNCaP cells were incubated in the presence of the IKBKE inhibitor CAY10576 for 24 hours prior to counting, resuspension in basal media and transfer to Boyden chambers. Chambers were incubated for 16 h prior to cell fixation, staining and counting. Chambers were set up in triplicate per condition. Data shown is expressed as mean cell number ± SD for 1 representative experiment of two independent experiments. (**F**) LNCaP cells were transfected in full media with either N/S or three pooled siRNAs against IKBKE. After 72 h, cells were re-seeded at a density of 2000 cells per well and cultured for 2 weeks. Colony forming efficiency was assessed by fixing and staining colonies with crystal violet (*n* = 3). Student's *t*-test **** *P* < 0.0001.

We next assessed the role of IKBKE in regulating the survival and proliferation of PC cell lines. To examine the phenotypic effect of IKBKE depletion on cellular growth, the proliferative potential of LNCaP cells was assessed following transfection with either N/S control or individual IKBKE siRNAs, using live cell imaging. In the IKBKE depleted cells, proliferation began to be impaired at around 54 h post transfection and was significantly reduced by 120 h (*P* < 0.05), confirming that IKBKE is required for normal PC cell growth (Figure 7A). As IKBKE knockdown results in AR inhibition from ∼48 h (data not shown) we speculate that this reduction could be a consequence of reduced AR levels. Interestingly, IKBKE knockdown in AR negative PC3 cells also reduced proliferation (Supplementary Figure S6A) suggesting that IKBKE can also influence proliferation independent of AR. However, it does appear that they are less sensitive to IKBKE knockdown when proliferation rates are compared. This is particularly apparent for siRNA #2. Additionally, cell cycle distribution evaluated using flow cytometry demonstrated a significant 19% increase of G1 phase cells alongside a significant 63% decrease of cells in S phase and a 53% decrease in G2/M phase in the IKBKE depleted cells, suggesting that IKBKE depletion results in a G1 cell cycle arrest (Figure 7B). This profile is similar to cell cycle distribution upon AR knockdown in the LNCaP cells line as AR has been described as a ‘master regulator’ of the transition between G1 and S phase (37) further implicating links between IKBKE and AR. Moreover, no increase in the proportion of cells in sub-G1 was evident, suggesting that IKBKE depletion is cytostatic rather than cytotoxic. In agreement with IKBKE depletion, pharmacological antagonism of IKBKE in multiple PC cell lines, resulted in a significant and dose dependent decrease in cellular growth, as assessed by live cell imaging (Figure 7C) or alternatively sulforhodamine B growth assays (Supplementary Figure S6B–D). Furthermore, AR positive cell lines appear more sensitive to IKBKE inhibition upon comparison of GI50 values (Supplementary Figure S6E–G) and ‘normal’ prostate cancer cells appear to be less sensitive (Supplementary Figure S6H).

As high IKBKE expression was previously reported to be associated with the development of bone metastases in PC patients (38) we next sought to determine the migratory ability of LNCaP cells following IKBKE suppression. IKBKE knockdown was carried out in serum containing media for 72 h prior to equal cell numbers being resuspended in serum-free media and transferred into Boyden chambers inserted into serum containing media as a chemoattractant. After 16 h cells were fixed, stained and counted to reveal a significant 91.7% reduction in migration. (Figure 7D). Similarly, upon IKBKE inhibition for 24 h prior to seeding into migration chambers using the same protocol a significant 82.4% reduction in migration was observed (Figure 7E). Furthermore, in both LNCaP and LNCaP-AI cells, IKBKE knockdown resulted in a significant 20-fold decrease in colony forming ability compared with N/S controls (Figure 7F and Supplementary Figure S6I, J). Additionally, IKBKE depletion prior to re-seeding significantly retarded cellular adhesion, a phenomenon which may prove therapeutically beneficial in preventing the engraftment and colonization of metastatic cells (Supplementary Figure S6K). Taken together these results suggest that diminishing IKBKE signalling in PC cells may prove therapeutically beneficial for patients by suppressing AR signalling to prevent cancer cell growth and metastases.

### IKBKE is required for endocrine therapy-resistant cell growth

**Figure 8.**
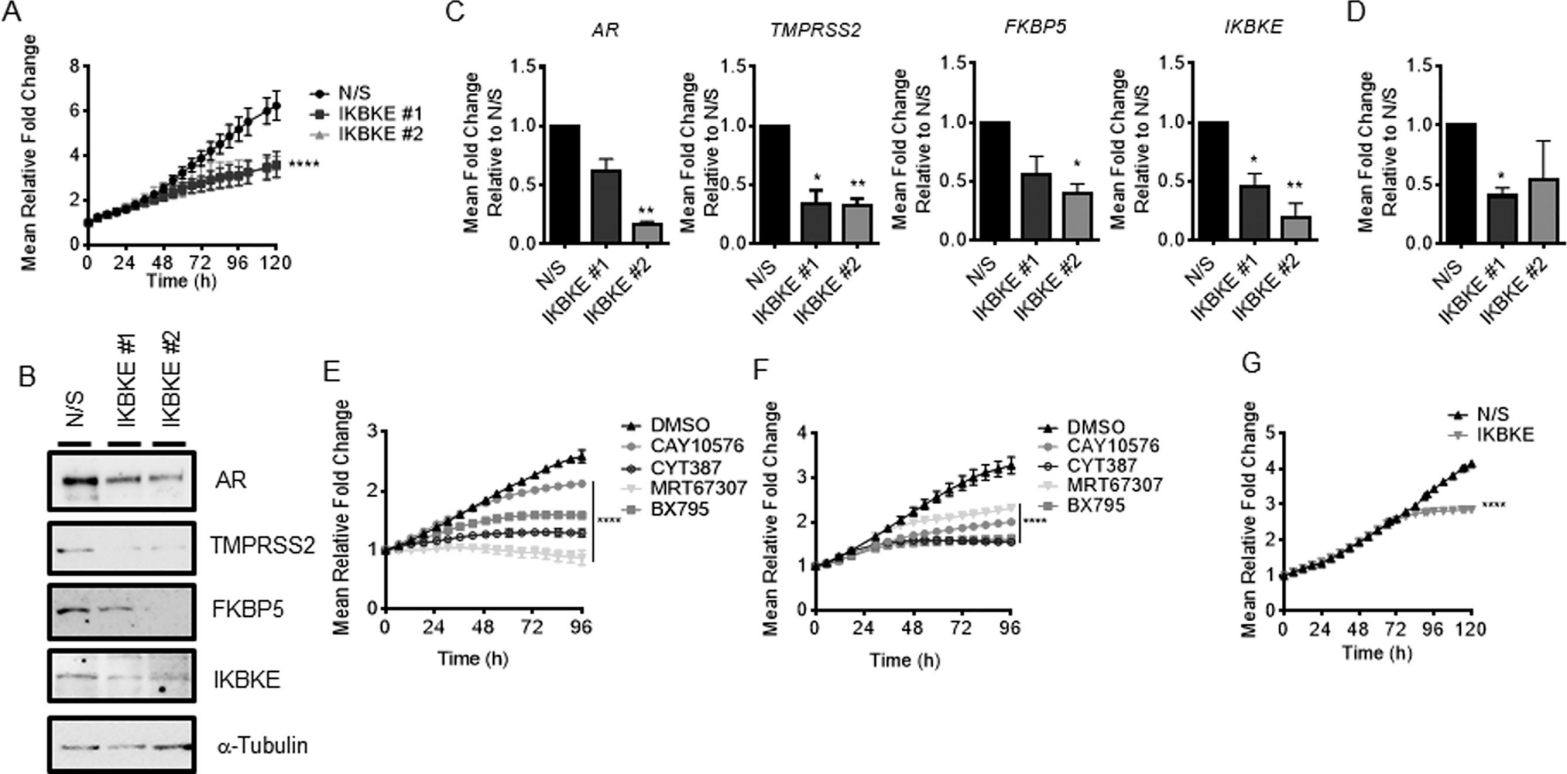
IKBKE is required for AR-target gene expression and cell growth in treatment-resistant PC cells. (**A**) LNCaP-EnzR cells were reverse transfected in full media with either N/S or two independent siRNAs against IKBKE and cell confluency assessed using the IncuCyte Zoom Live Cell Imager every 6 h for 96 h (*n* = 3). (**B**) LNCaP-EnzR cells were treated as in (A) after 96 h, AR, TMPRSS2, FKBP5 and IKBKE expression was determined by immunoblotting and (**C**) *AR, TMPRSS2, FKBP5* and *IKBKE* mRNA expression determined by qPCR (*n* = 3). (**D**) LNCaP-EnzR cells were transfected in full media with either N/S or three pooled siRNAs against IKBKE. After 72 h, cells were re-seeded at a density of 2000 cells per well and cultured for 2 weeks. Colony forming efficiency was assessed by fixing and staining colonies with crystal violet (*n* = 3). (**E**) LNCaP-EnzR cells were seeded and left to adhere and grow for 24 h, prior to treatment with either vehicle (DMSO) or the IKBKE antagonists, CAY10576 (5 μM), BX795 (5 μM), MRT67307 (5 μM), or CYT387 (5 μM) and cell confluency assessed using the IncuCyte Zoom Live Cell Imager every 6 h for 96 h (*n* = 2). (**F**) CWR22Rv1 cells were seeded and left to adhere and grow for 24 h, prior to treatment with either vehicle or CAY10576 (5 μM), BX795 (5 μM), MRT67307 (5 μM), or CYT387 (5 μM) and cell confluency assessed using the IncuCyte Zoom Live Cell Imager every 6 h for 96 h (*n* = 2). (**G**) CWR22Rv1 cells were reverse transfected with either N/S or three pooled siRNAs against IKBKE, in full media supplemented with 10 μM Enz and cell confluency assessed using the IncuCyte Zoom Live Cell Imager every 6 h for 120 h (*n* = 3). 2way ANOVA, Dunnett's multiple comparisons test used in A, E, F and G **** *P* < 0.0001. One-way ANOVA paired Dunnetts multiple comparison test used in C and D * *P* < 0.05, ** *P* < 0.001.

Identifying novel therapeutic targets within the AR signalling cascade is essential to overcome endocrine therapy-resistant disease. As high IKBKE expression is correlated with castration resistance (38) we therefore next sought to investigate the effect of IKBKE depletion and pharmacological antagonism on endocrine therapy-resistant PC cell growth. The LNCaP-enzalutamide resistant cell line, LNCaP-EnzR and the CWR22Rv1 cell line both represent models of endocrine therapy-resistant disease, in which cells continue to grow and respond to androgenic stimuli in the presence of the anti-androgen enzalutamide. Proliferation tracking upon IKBKE knockdown revealed a significant 44% reduction in proliferation at 120 h in LNCaP-EnzR cells (*P* < 0.05) (Figure 8A) which was consistent with cell counts (Supplementary Figure S7A). Furthermore, analysis of *AR*, *TMPRSS2* and *FKBP5* mRNA and protein expression showed that AR signalling was significantly inhibited (Figure 8B,C). Colony forming ability was also reduced upon IKBKE knockdown in this cell line (Figure 8D). A similar repression of proliferation was apparent with all IKBKE small molecule inhibitors in both LNCaP-EnzR (Figure 8E) and CWR22Rv1 (Figure 8F) cells. Moreover, IKBKE knockdown was found to still inhibit the growth of CWR22Rv1 cells in the presence of Enzalutamide (Figure 8G) indicating that IKBKE can also influence AR variant facilitated cell growth. Furthermore, both full length and variant AR proteins showed decreased expression in VCaP cells upon IKBKE knockdown in the presence of enzalutamide (Supplementary Figure S7B). The data therefore indicates that IKBKE continues to regulate AR signalling in cells which have developed endocrine therapy resistance and therefore pharmacological antagonism of IKBKE could potentially be used to treat both primary and endocrine therapy-resistant disease.

### IKBKE is therapeutically targetable in PC *in vivo*

**Figure 9.**
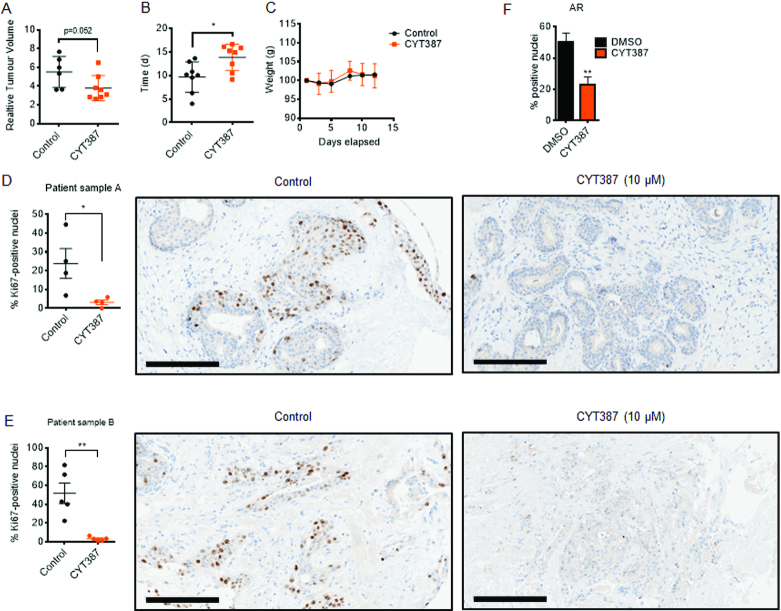
IKBKE inhibition reduces tumour volume and inhibits proliferation of PC *ex vivo* cultures. CWR22Rv1 cells were implanted subcutaneously in nude mice, and following the development of established xenograft tumours, vehicle (DMSO) (*n* = 6) or CYT387 100 mg/kg (*n* = 8) was administered daily by oral gavage. (**A**) Relative Tumour Volume (compared to dosing day 0) ± SD following either 12 days vehicle or CYT387 treatment. (**B**) Time for CWR22Rv1 xenografts to reach 4× Relative Tumour Volume (compared to dosing day 0) ±SD following daily vehicle or CYT387 treatment. (**C**) Mean murine body weight over time, following daily vehicle or CYT387 treatment. (**D**, **E**) *Ex vivo* cultures of two human PC tumours (patient sample A (chTURP); patient sample B (RRP)) were established and treated with either vehicle or IKBKE inhibitor CYT387 (10 μM) for 48 h. Expression of the proliferative marker Ki67 was then assessed in treated explant tissue sections by immunohistochemistry and digitally quantified. Data is expressed as percentage of cells positively stained for Ki67. Representative images of Ki67 staining are shown. Mann–Whitney *U* test; two-tailed: *P < 0.05, ** *P* < 0.01. Data points represent the percentage of Ki67-positive nuclei within a representative 40× magnification field, with a total of at least 1000 nuclei analysed for each treatment. Error bars indicate the mean ± SEM. Scale bars = 200 μm. (**F**) A third *ex vivo* culture was treated as in D and E but also assessed for AR expression alongside AMACR (cancerous marker) and p63 (benign marker). AR staining was then scored in AMACR positive glands as described above. chTURP, channel transurethral resection of the prostate; RRP, robotic radical prostatectomy.

The efficacy of IKBKE pharmacological antagonism *in vivo* was tested using the Phase III, dual IKBKE/JAK2 antagonist, CYT387, on CWR22Rv1 tumour xenograft growth. This inhibitor was favoured over CAY10576 as it has superior chemical properties for *in vivo* studies. As this inhibitor also has JAK2 activity we first compared the growth of CWR22Rv1 cells *in vitro*, in the presence of either CYT387 or a specific JAK2 inhibitor, Ruxolitinib. This showed that JAK2 inhibition does not inhibit the growth of CWR22Rv1 cells (Supplementary Figure S7C) hence any effect of CYT387 in CWR22Rv1 cells can be attributed to inhibition of IKBKE and not JAK2. After tumours were established in immunodeficient mice (average tumour volume 100 mm^3^), CYT387 was administered via daily oral gavage at a dose of 100 mg/kg. Compared with a vehicle control, CYT387 treatment at this dose effectively inhibited tumour growth (Figure 9A) and significantly delayed tumour progression (Figure 9B), with no deleterious effects on murine body mass (Figure 9C). We next explored the therapeutic potential of IKBKE antagonism using CYT387 in a system that more closely recapitulates human tumour physiology using patient-derived prostate tissue explants. Similar to CWR22Rv1 xenografts, CYT387 treatment impaired the growth of explant prostate glandular tissue, as measured by the prevalence of Ki67 positive nuclei (Figure 9D, E; Supplementary Figure S8, Supplementary Table S4). Furthermore, we demonstrate that AR expression is reduced in response to CYT387 treatment (Supplementary Figures S9A, B). Taken together, these findings suggest that inhibition of IKBKE signalling in PC suppresses AR mediated transcriptional activation and cellular growth and the utilization of small molecule inhibitors of IKBKE may exhibit therapeutic potential for the treatment of both androgen sensitive and therapy resistant PCs.

## DISCUSSION

The AR remains a key therapeutic target in advanced, anti-androgen resistant disease. As an alternative means to reduce AR activity when anti-androgens have failed, co-regulator proteins which control the activity of the AR can be targeted. Indeed, many of these co-regulators are dysregulated in advanced disease (2). Ideally, co-activators with enzymatic activity could be targeted with small molecule inhibitors to indirectly diminish AR activity. As multiple kinase enzymes have been shown to play a key role in regulating AR activity and several clinically approved kinase inhibitors are available, we chose to investigate which protein kinases may provide the greatest clinical benefit to anti-androgen resistant patients. By interrogating the entire human kinome, we identified IKBKE as a potential AR regulating protein.

Here, we demonstrate that IKBKE plays a role in the production of AR mRNA and as such results in enhanced AR regulated gene expression. As IKBKE mediated AR regulation occurs at the level of transcription, all AR mRNA species are affected including those which result in the production of constitutively active splice variant forms for which there are currently no targeted therapeutics available. The kinase activity of IKBKE appears to be important in regulating AR mRNA production therefore, we hypothesize that inhibition of IKBKE may be therapeutically beneficial to CRPC patients who frequently retain expression of functional AR and AR-Vs. Indeed, we show that knockdown and inhibition of IKBKE can reduce proliferation, migratory ability and tumour volume supporting our hypothesis that IKBKE inhibition is a viable clinical strategy. Furthermore, it has been reported that high cytoplasmic IKBKE expression associates with the development of bone metastases and castration resistance (38) consistent with a role in therapy resistant disease. Indeed, treatment of enzalutamide resistant PC cell line models with either siRNA-mediated knockdown or pharmacological inhibition of IKBKE results in a robust inhibition of proliferation.

Currently, two compounds are available in the clinic that target IKBKE, each displaying minimal side effects; Amlexanox and CYT387. Amlexanox, an IKBKE/TBK1 inhibitor developed to treat ulcers, allergic rhinitis and asthma, results in a G0/G1 arrest in glioblastoma cells and induction of apoptosis (13). We also observed G0/G1 arrest following IKBKE knockdown in PC cells but the apoptosis rate was unaffected. Importantly, as Amlexanox has already been administered to humans in a clinical trial for Type 2 diabetes (22), repurposing this drug in PC patients who are resistant to AR targeting therapies could be tested quickly.

In order to determine the mechanism by which cytosolic IKBKE positively regulates AR mRNA we investigated transcription factors that have been shown to regulate the expression of AR and which functionally interact with IKBKE. Firstly, we ruled out NF-κB inactivation as p65 is cytoplasmic in LNCaP derivative cell lines. Secondly, c-MYC was investigated as a mediator between IKBKE and AR expression. Interestingly, we observed a decrease in c-MYC protein levels in response to IKBKE knockdown or pharmacological inhibition which resulted in decreased c-MYC binding to the AR gene. We confirmed that c-MYC down-regulation was at the transcriptional level and not due to increased c-MYC turnover. This contrasts with observations in pancreatic ductal adenocarcinoma cell lines where IKBKE mediated phosphorylation of Akt and subsequent inhibition of GSK3β regulates the nuclear retention and stabilization of c-MYC without impacting on c-MYC mRNA (14). However, these differences could possibly be explained by the presence of a constitutively active Akt and low GSK3β activity in LNCaP cells (39). In addition, during the preparation of this manuscript c-MYC was identified as a transcriptional and functional regulator of the splicing factor Sam68 (40) which has been shown to be a key factor in the production of AR splice variants (41,42) further supporting a role for IKBKE, as an upstream regulator of c-MYC, in driving CRPCs.

Numerous studies describe c-MYC as transcriptionally regulated by YAP, a component of the Hippo pathway (13,43,44). As YAP has been widely studied in the context of PC we investigated further and found that IKBKE knockdown or inhibition reduced the total and nuclear levels of YAP supporting a role for YAP in this mechanism. Conversely, we observed that IKBKE knockdown increased YAP mRNA expression suggesting that the observed decrease in protein level is due to an increase in YAP turnover. Indeed, enhanced YAP turnover via the ubiquitin-mediated proteasomal pathway has been reported as a consequence of IKBKE mediated phosphorylation and activation of LATS (13). Consistent with this, we observed that IKBKE knockdown resulted in enhanced YAP proteasomal degradation which was rescued by the addition of the proteasomal inhibitor MG132. Furthermore, it is evident that the rate of YAP turnover dominates over the increased YAP mRNA levels which is a consequence of relieved YAP promoter methylation normally facilitated by AR interacting with EZH2 and DNMT1 (43).

Whilst IKBKE regulation of AR mRNA production appears to be the primary mechanism of control, we have evidence that IKBKE affects AR transcriptional activity independently of *de novo* protein synthesis. Upon knockdown of IKBKE in LNCaP cells engineered to constitutively over express a GFP-tagged AR driven by a CMV promoter, no alterations in AR-GFP protein levels were observed, however PSA levels were robustly depleted. Furthermore, AR-GFP recruitment to the PSA enhancer was significantly reduced. One potential mechanism whereby IKBKE regulates AR protein activity may be via IKBKE-mediated AKT phosphorylation at Thr308/ Ser473 sites. Activated pAKT can directly phosphorylate AR to promote activation (45) or indirectly act via GSK3β Ser9 phosphorylation that subsequently causes AR activation (46). The IKBKE-targeted LATS and YAP proteins are also reported to impact AR activity (34,47). Furthermore, we observed that AR positive cell lines were more sensitive to IKBKE inhibition than AR null cell lines. Hence, multiple links exist between increased expression of IKBKE and enhanced AR transcriptional activity.

With links established between IKBKE gene amplification and AR independent neuroendocrine PCs and our data regarding IKBKE knockdown and PC3 cellular proliferation we acknowledge that there is a potential for AR independent effects. Indeed, IKBKE expression in PC3 cells is much higher than in LNCaP cells. Interestingly, upon knockdown of c-MYC in PC3 cells a robust inhibition of cellular proliferation is observed which would explain why IKBKE knockdown, which decreases c-MYC levels, has this effect. Whilst this requires further investigation, there is also scope to investigate the utility of IKBKE targeting therapeutics in highly aggressive AR negative tumours.

**Figure 10.**
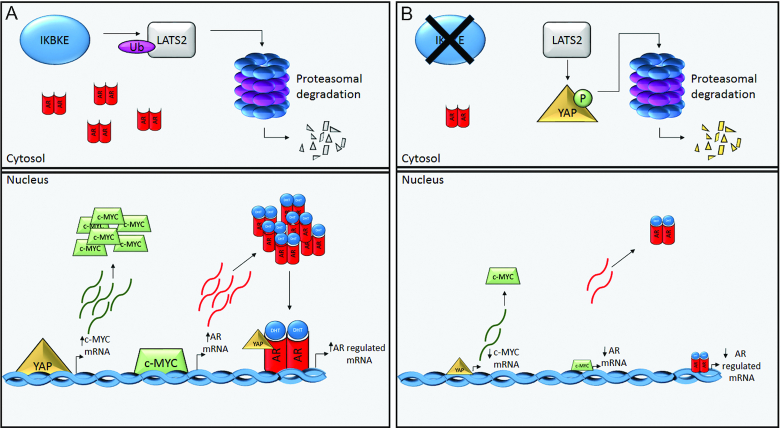
Model of IKBKE mediated AR signalling. (**A**) IKBKE up-regulation in prostate cancer enhances the ubiquitination and turnover of LATS2 in a kinase dependent manner. This is turn promotes nuclear localization of YAP and transcription of YAP regulated genes, including c-MYC. Up-regulated levels of nuclear c-MYC result in up-regulation of c-MYC regulated genes, such as AR. This results in a large pool of AR in cells which in turn, upon ligand mediated activation can also be co-activated by nuclear YAP. (**B**) Upon inhibition of IKBKE kinase activity, LATS2-Ub is impaired allowing the protein to accumulate and phosphorylate YAP. Phosphorylated YAP is held in the cytoplasm and subsequently targeted for proteasomal degradation. This results in c-MYC downregulation which in turn results in AR down-regulation, including constitutively active AR splice variants, and therefore AR regulated gene expression. Ub, ubiquitin; P, phosphate.

In summary, this study demonstrates that IKBKE plays a role in AR signalling. Small molecule inhibitors of IKBKE kinase function diminishes AR signalling by simultaneous downregulation of AR mRNA and transcriptional activity. This downregulation is a result of IKBKE crosstalk with the Hippo pathway resulting in LATS2 upregulation, which in turn downregulates YAP by phosphorylation-mediated nuclear exclusion and degradation, to deplete its transcriptional target c-MYC which is important in the production of AR mRNA (Figure 10). The inhibition of this signalling pathway results in favourable phenotypic outcomes linked to cancer therapeutics such as reduced AR regulated gene expression, decreased migratory ability, reduced tumour volume and reduced proliferative potential. As YAP is reported to be upregulated in CRPC and to be responsible for androgen-independent growth, we propose that targeting IKBKE will help to overcome YAP-mediated effects and be of particular benefit to patients who develop resistance to current AR targeting therapies.

## Supplementary Material

gkaa271_Supplemental_File
