# Supplementary material for: IKBKE activity enhances AR levels in advanced prostate cancer via modulation of the Hippo pathway
Source: Nucleic Acids Res. 2020 Apr 23;48(10):5366–82. doi: 10.1093/nar/gkaa271 (PMC7261174; doi:10.1093/nar/gkaa271)
Supplement: gkaa271_Supplemental_File [file gkaa271_supplemental_file.pdf]

Supplementary Materials and Methods

Antibodies

AMACR (2AF10F3) sc-81710, p63 (D-9) sc-25268, NFκB p65 (C-20) sc-372 (Santa Cruz Biotechnologies)

Generation of LNCaP-AI AREIII-LUC cell line

Briefly, PSA85(AREIII)3-4-Luc and a Zeocin resistant plasmid was obtained from Prof Jan Trapman (EMC, Rotterdam). Both constructs were co-transfected into LNCaP-AI cells using TransIT™-LT-1 (Mirus) and maintained under zeocin selection until colonies formed. Individual colonies were then picked and expanded. Colonies were then screened by luciferase assay after stimulation with synthetic androgen, R1881 (1 nM), to induce AR activity. From this, clones were selected and demonstrated to induce a luciferase signal in response to R1881 treatment. Comparisons were then made between the clones and the LNCaP-7B7 cell line so that upon siRNA screening base line signals between the LNCaP-7B7 and LNCaP-AI clone were comparable. A single clone was used throughout all the experiments.

Lentiviral expression and LNCaP GFP-AR cell line generation

The coding sequence for GFP-AR (1) was sub-cloned in-frame into the pCDH-EF1-FHC lentiviral transfer vector, purchased from Addgene, using the restriction enzymes Nhe1 and Xba1. Gene reporter assays were carried out to confirm AR functionality in PC3 cells which do not express endogenous AR. Stably expressing LNCaP cells were generated by lentiviral infection, using the protocol described in Walker *et al* (2). Briefly, HEK293T cells were co-transfected using TransIT™-LT1 reagent (Mirus) with pCDH GFP-AR, pPsPax2 (Addgene), pMD2G (Addgene), according to manufacturer's instructions. Virus containing media was subsequently collected 24-48 hr post transfection, sterile filtered, and applied to parental LNCaP cells in 8 μg/ml Polybrene (Millipore).

LNCaP Yap S127A cell line generation

The coding sequence of Yap with Serine 127 mutated to Alanine was obtained from Addgene plasmid pCMV-flag S127A YAP (#27370). Due to a high GC content problems were encountered during cloning procedures which prevented direct cloning into pLenti-puro (Addgene #39481) via PCR based methods. Therefore, we generated a gBlock (IDT) containing this sequence, with GC rich areas minimised by codon switching, and also contained the restriction enzyme recognition sites to allow cloning to be performed (Supplementary Figure 10). Both gBlock and pLenti-puro were digested with Spe1 and EcoRI and then ligated. DNA was then amplified in E.coli and positive colonies selected for screening. Following sequencing of selected colonies a selection were transfected into HEK293T cells alongside control vectors to confirm that Yap is expressed as expected and that the codon switching has not impacted on protein production. Yap-S127A was clearly detected using both the V5 antibody and the Yap antibody confirming effective expression from the generated plasmid (Supplementary Figure 11). Then, stably expressing LNCaP cells were generated by lentiviral infection as described above. Briefly, HEK293T cells were co-transfected using TransIT™-LT1 reagent (Mirus) with pLenti-puro-Yap S127A or empty vector control, pPsPax2 (Addgene), pMD2G (Addgene), according to manufacturer's instructions. Virus containing media was subsequently collected 24-48 hr post transfection, sterile filtered, and applied to parental LNCaP cells in 8 μg/ml Polybrene (Millipore). Puromycin selection was applied to the cells 48 h post-transduction.

siRNA kinome library screen

For high-throughput screening, cells were reverse transfected in 384 well, white, clear bottomed plates (Greiner) using RNAiMax (Invitrogen). In brief, siRNAs were prealiquoted in 384-well plates to give a final concentration of 25 nM using a Biomek® FXP Laboratory Automation Workstation (Beckman Coulter). RNAiMax was diluted in basal media supplemented with 2 mM L-Glutamine and complexed with the siRNAs for 30 min. LNCaP-

7B7 and LNCaP-AI AREIII-LUC cells were seeded (5,000 cells/well) in steroid depleted culture medium over the precomplexed siRNA/RNAiMAX. After 72 h, cells were stimulated with either vehicle or R1881 (1 nM) and incubated for 24 h. To account for variation in LUC output as a consequence of cell confluency, individual well cell confluency was assessed using the IncuCyte ZOOM prior to LUC assay implementation. To assess LUC activity, Steady Glo Luciferase Reagent (Promega) was added directly to cell culture media at a ratio of 1:6. Luminescence signal (RLU) was measured using a FLUOstar Omega plate reader (BMG Labtech), with RLU readings being normalised to individual well confluency. Primary screening was performed using Dharmacon Human Drugable Genome Library (protein kinases and associated proteins only) Top 20 candidate AR activators were selected for further investigation from all arms of the primary screen. Putative AR activators which were common to the top 100 kinases in all experimental arms were also included. Secondary screening was performed on these kinases using pooled independent siRNA sequences from the Sigma Kinome Screening Library.

Determination of proliferative potential using cell counts

For cell counts, cells were transfected as described above and grown for 96 h prior to imaging at least nine fields per well using the IncuCyte ZOOM. Images were subsequently quantified using FIJI analysis software (3). Data were presented as the mean cell number from three independent experiments +/- SEM.

IKBKE Amplification Status Data Analysis

Amplification data from 19 Studies held within cBioportal (4,5) were investigated for IKBKE amplification status totalling 4979 patients/5260 samples. The following studies were included: Prostate Cancer (MSK, 2019), The metastatic prostate cancer project. (The results included here include the use of data from The Metastatic Prostate Cancer Project (https://mpcproject.org/), a project of Count Me In (https://joincountmein.org/)), (6-20).

Immunohistochemistry

Immunohistochemistry was performed as described in the main manuscript with the following amendments. AMACR primary antibody (1:100), AR (1:500) and p63 (1:100) were incubated for 1 h at 37°C. Stained slides were imaged using an Aperio CS2 Digital Pathology scanner (Leica). Areas which were AMACR positive were then scored for AR nuclear staining using Aperio Image Analysis Software (Leica) and data presented as % positive nuclei within this region. 12 regions of interest were analysed per treatment. No primary controls are shown for positive staining and positive controls for negative staining are shown in Supplementary Figure 9C.

Supplementary Figures

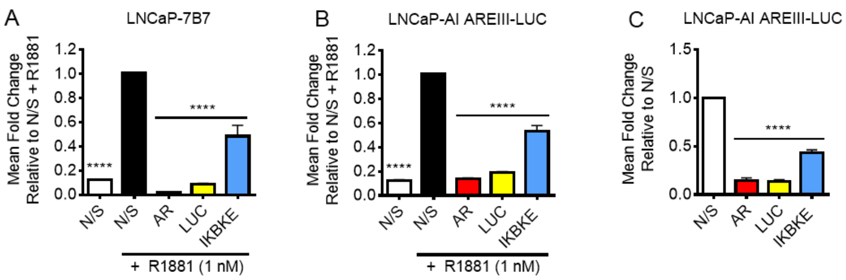

Supplementary Figure 1. IKBKE is an AR regulator. (A) LNCaP-7B7 and (B) LNCaP-AI AREIII-LUC were reverse transfected in steroid depleted media (SDM) with siRNAs targeting either N/S, AR, LUC or IKBKE (pool of 3 independent siRNA sequences) for 72 h. R1881 (1 nM) was then applied for a further 24 h. Luciferase activity was measured and normalised to cell confluency. (C) LNCaP-AI AREIII-LUC cells were treated as in (B) except no R1881 stimulation was applied. Luciferase activity was then measured and normalised to cell confluency. Data is expressed as mean fold change relative to N/S control + R1881 +/- SEM. One-way ANOVA **** $p<0.0001$

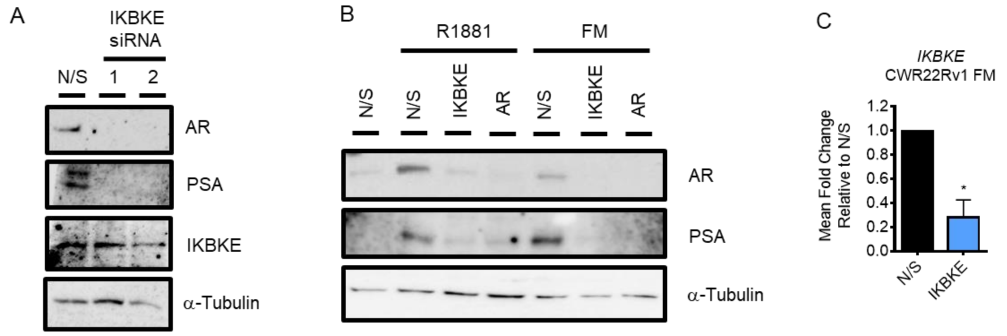

Supplementary Figure 2. IKBKE regulates AR expression. (A) LNCaP-AI cells were transfected in steroid depleted media (SDM) with N/S or two individual siRNAs targeting IKBKE. After 72 h, PSA, AR and IKBKE expression was assessed by immunoblotting. (B) LAPC4 cells were transfected with N/S, AR targeting siRNA or pooled IKBKE siRNAs and grown in either SDM + R1881 (1 nM) or FM. After 72 h, PSA and AR expression was determined by immunoblotting. (C) CWR22Rv1 were transfected with N/S or pooled IKBKE siRNAs and grown in FM. 72 h later cells were lysed and assessed for *IKBKE* expression by qPCR (n=3). Student t-test * $p<0.005$

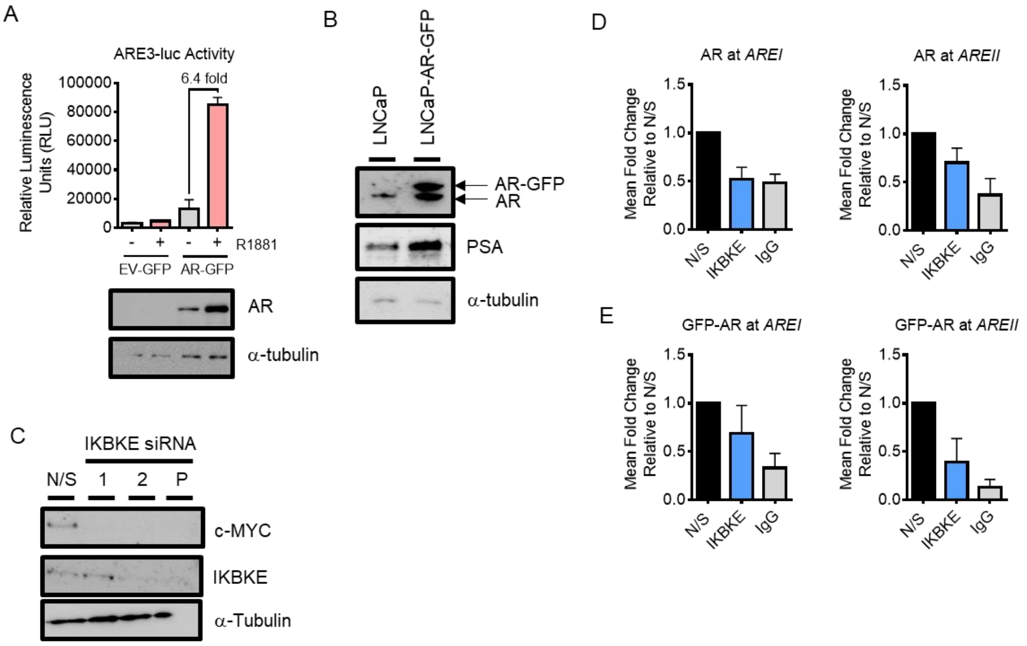

Supplementary Figure 3. LNCaP-AR-GFP constitutively expresses a functional GFP tagged AR. (A) Gene reporter assays performed using AREIII-Luc transiently transfected into AR null PC3 cells in the presence or absence of AR-GFP confirmed AR-GFP functionality. (B) Equal cell numbers or the parental LNCaP and the LNCaP-AR-GFP cell lines were lysed and analysed by western blotting for the expression of AR, AR-GFP and the androgen receptor regulated PSA protein. (C) IKBKE knockdown confirmation for samples used to generate Figure 3, alongside levels of c-MYC. (D) Chromatin immunoprecipitation using either anti-AR antibody, (E) anti-GFP antibody or IgG control was performed and recruitment to the PSA promoter/enhancer (AREI and AREII) was assessed by qPCR (n=3). Data is expressed as mean fold change relative to N/S control +/- SEM.

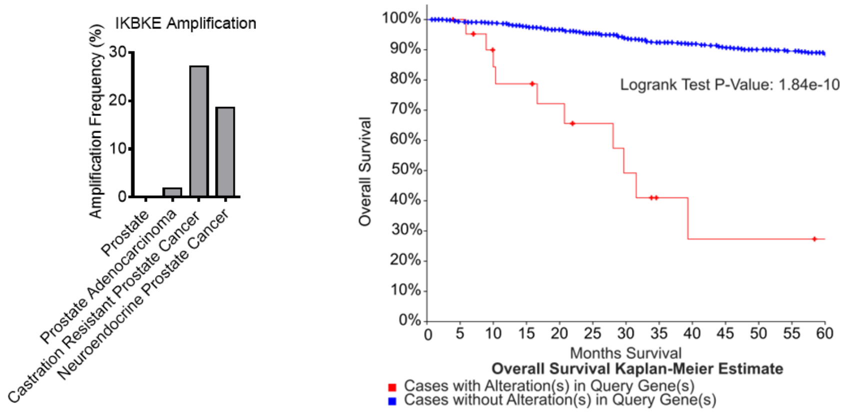

Supplementary Figure 4. IKBKE Amplification is associated with disease progression and patient survival. Prostate cancer datasets within cBioportal were analysed for IKBKE alterations revealing that IKBKE is (A) amplified in ~27% of cases of castration resistant prostate cancer (19/70 cases) compared to 1.74% (86/4711 cases) in prostate adenocarcinoma and that (B) alteration in IKBKE are associated with poorer patient survival.

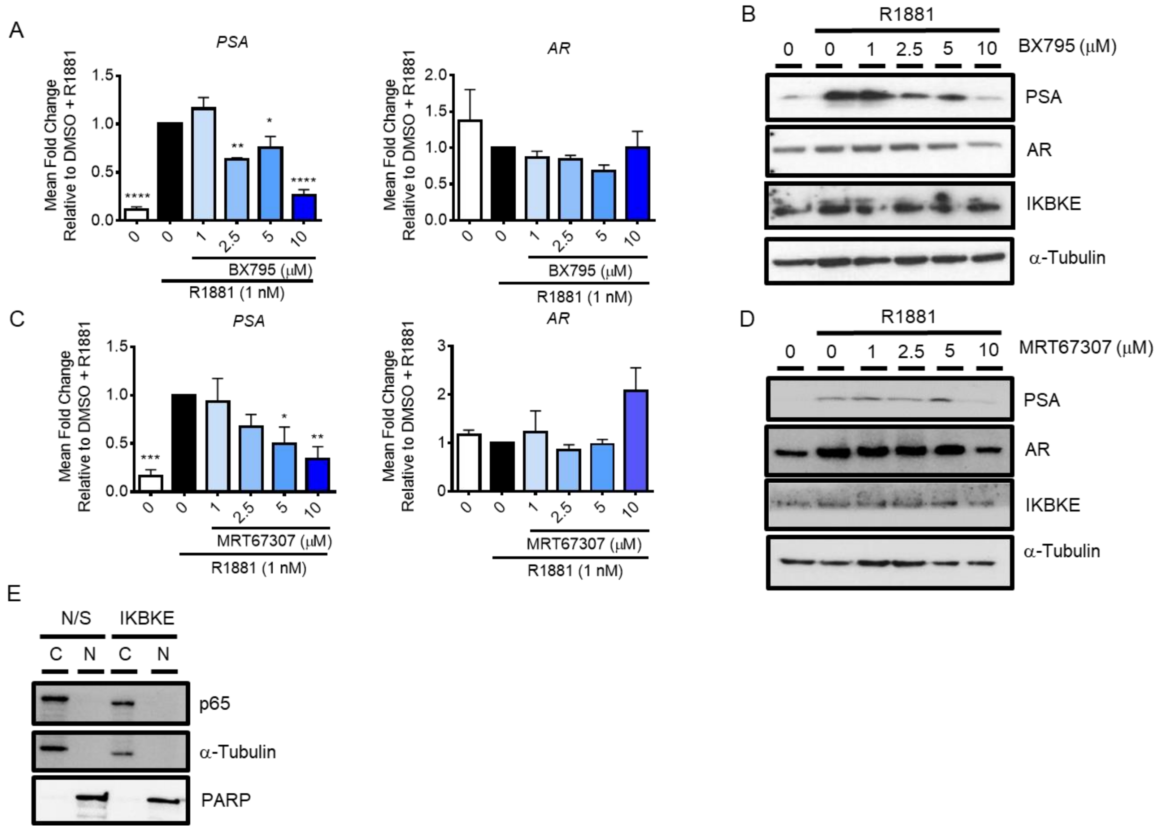

Supplementary Figure 5. IKBKE regulates AR mRNA. After 72 h steroid depletion, LNCaP cells were pre-treated with vehicle (DMSO) or increasing concentrations of (A) BX795 or (B) MRT67307 for 8 h followed by treatment with vehicle (DMSO) or 1 nM R1881 for 24 h. *PSA* and *AR* mRNA expression determined by qPCR (n=3) and (C, D) PSA and AR protein expression was determined by immunoblotting. Unpaired one-way ANOVA, Dunnett's multiple comparisons test * $p<0.05$; ** $p<0.01$, *** $p<0.001$, **** $p<0.0001$. (E) LNCaP cells were reverse transfected in full media (FM) with either N/S or three pooled siRNAs against *IKBKE*. After 72 h, nuclear-cytoplasmic fractionation was performed with p65 localisation determined by immunoblotting. PARP was used as a nuclear loading control and α-tubulin as a cytoplasmic loading control. Western blots are representative of at least 2 experimental repeats. α-tubulin was used as a loading control.

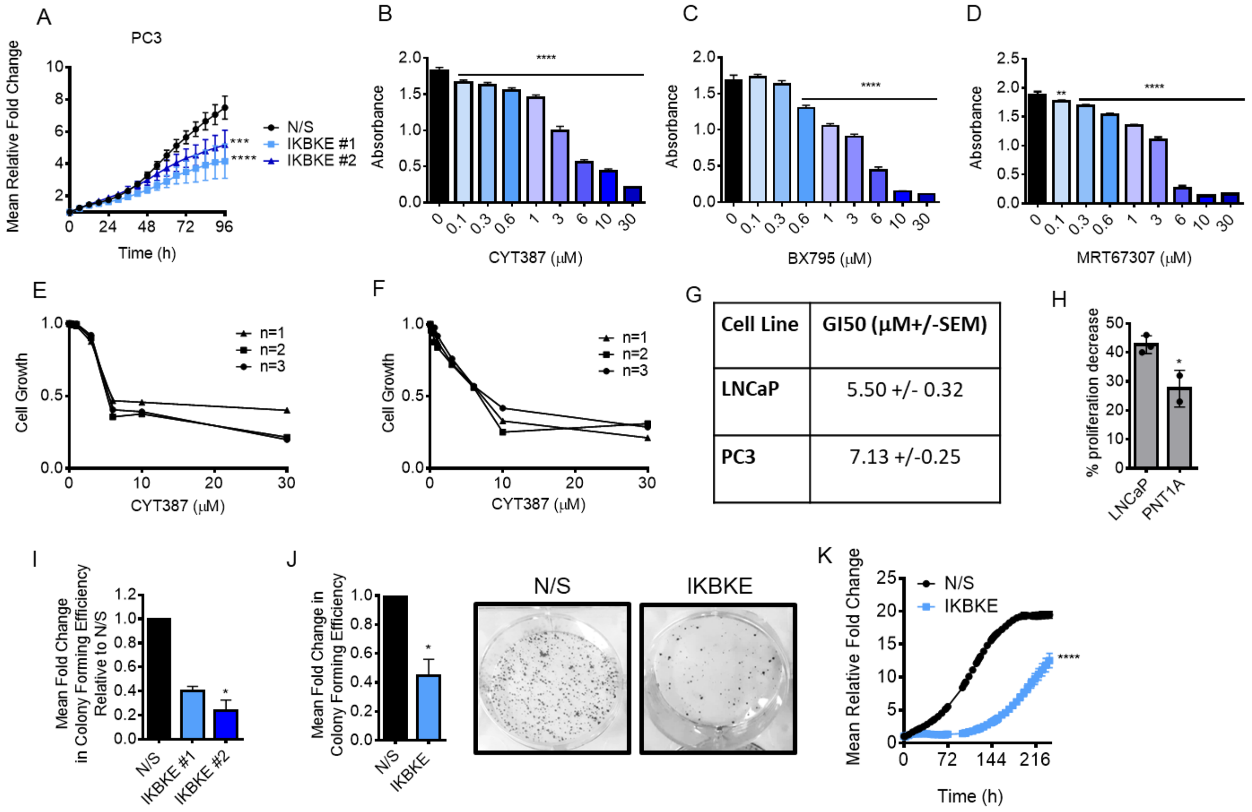

Supplementary Figure 6. IKBKE regulates prostate cancer cell proliferation. (A) PC3 cells were reverse transfected in full media with either N/S or two independent siRNA sequences targeting IKBKE and cell confluency assessed using the Incucyte Zoom Live Cell Imager every 6 h for 96 h (n=3). 2way ANOVA, Dunnett's multiple comparisons test *** p<0.001; **** p<0.0001. (B) LNCaP cells were incubated for 24 h in FM prior to addition of increasing doses of CYT387, (C) BX795 or (D) MRT67307. After 96 h, cellular proliferation was determined on 6 replicates per concentration by SRB assay (n=1). (E) LNCaP cells and (F) PNT1A cells were treated with increasing doses of CYT387 for 3 doubling times prior to fixation and analysis using SRB assays to determine GI50 concentrations (G) using GraphPad Prism software. (H) PNT1A cells were grown in the presence of CYT387 for 3 doubling times prior to SRB assay. Percentage reduction in proliferation at 5mM CYT387 was determine using GraphPad Software and compared to LNCaP cells grown under the same conditions. (I) LNCaP cells were transfected in full media with either N/S or two independent siRNAs against IKBKE. After 72 h, cells were re-seeded at a density of 2000 cells per well and cultured for 2 weeks. Colony forming efficiency was assessed by fixing and staining colonies with crystal violet (n=3). (J) LNCaP-AI cells were reverse transfected in SDM with N/S or IKBKE (pool). After 72 h cells were reseeded and cultured for 2 weeks. Colony forming efficiency was assessed by fixing and staining colonies with crystal violet (n=3). Paired, Student's t-test * p<0.05. (K) LNCaP cells were reverse transfected in FM with either N/S or IKBKE (pool) and incubated for 72 h. Cells were then re-seeded and cell attachment monitored over time using Incucyte. Data is expressed as mean fold change of independent experiments +/- SEM. 2way ANOVA, Dunnett's multiple comparisons test **** p<0.0001.

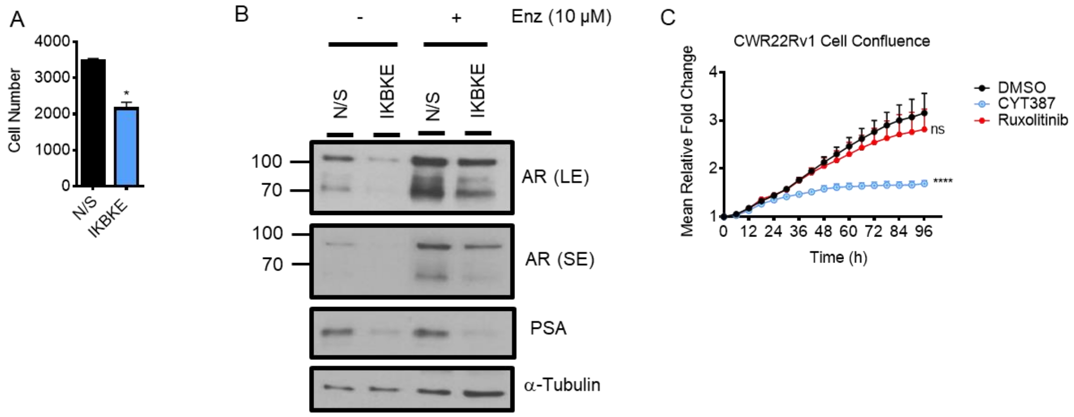

Supplementary Figure 7. IKBKE is a therapeutic target in therapy resistant PC. (A) LNCaP-EnzR cells were reverse transfected in full media with either N/S or three pooled siRNAs against IKBKE. After 96 h cells were trypsinised and counted (n=3). Data expressed as mean +/- SEM. Paired Student's t-test * $p<0.05$. (B) VCaP cells were reverse transfected in full media with either N/S or three pooled siRNAs against IKBKE. After 72 h, enzalutamide was applied for a further 24 h. AR and PSA expression was determined by western blotting. (C) CWR22Rv1 cells were treated with either DMSO, CYT387 (5 μM) or the JAK2 inhibitor, Ruxilitinib (5 μM). Cell confluence was tracked over time using Incucyte (n=2). 2way ANOVA, Dunnett's multiple comparisons test **** $p<0.0001$. LE = long exposure; SE = short exposure.

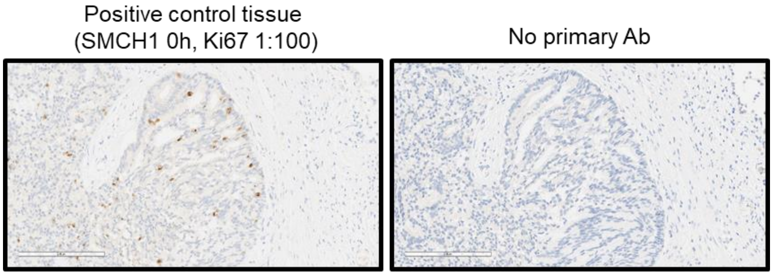

Supplementary Figure 8. Control staining for Ki67.

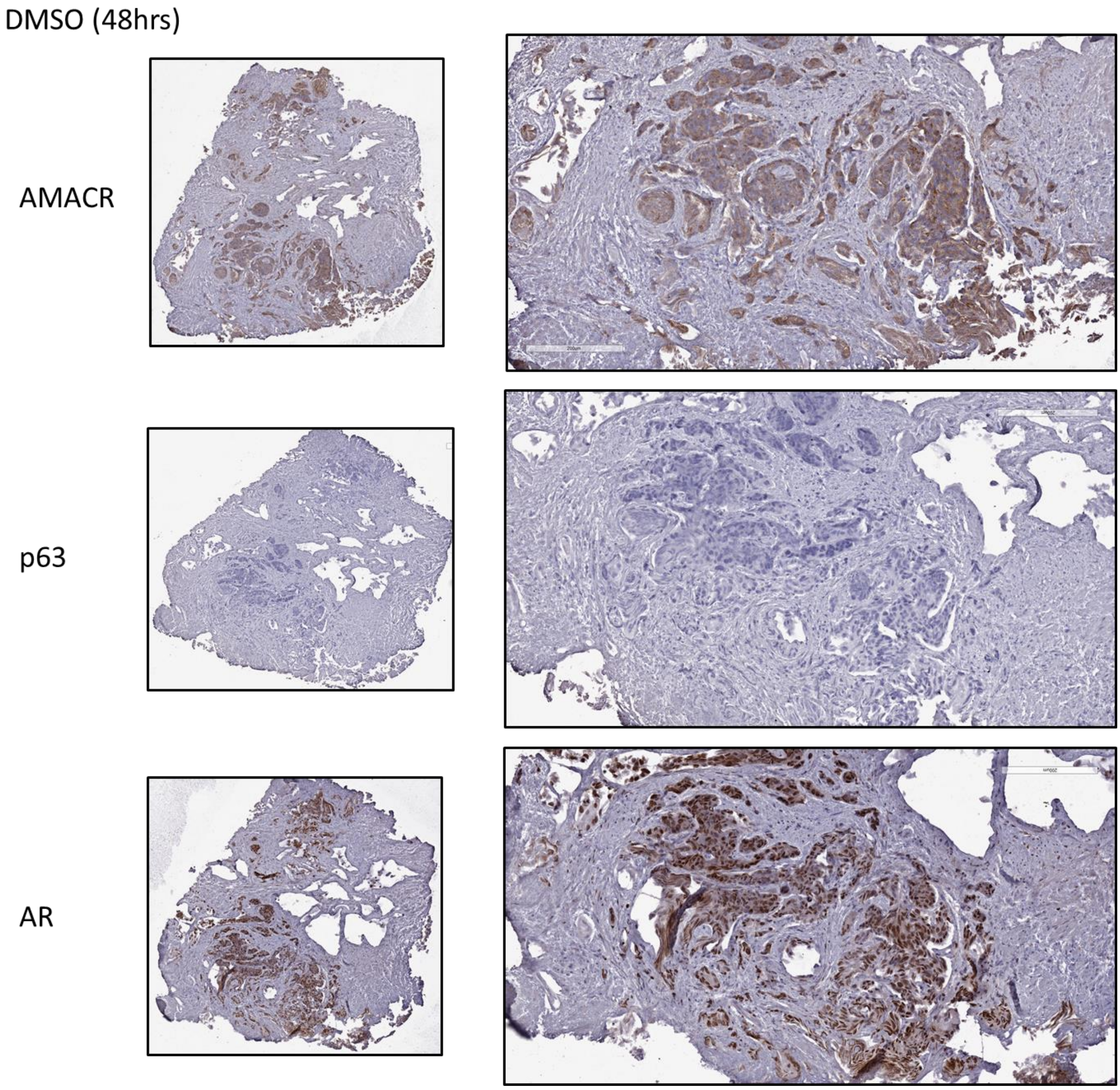

Supplementary Figure 9A. Representative images of pharmacodynamic analysis in *Ex vivo* cultures of human prostate cancer. *Ex vivo* culture of a human prostate cancer was established and treated with vehicle control (DMSO) for 48 h. Expression of AMACR (malignant marker), p63 (benign marker) and AR was assessed by IHC.

CYT (48 hrs)

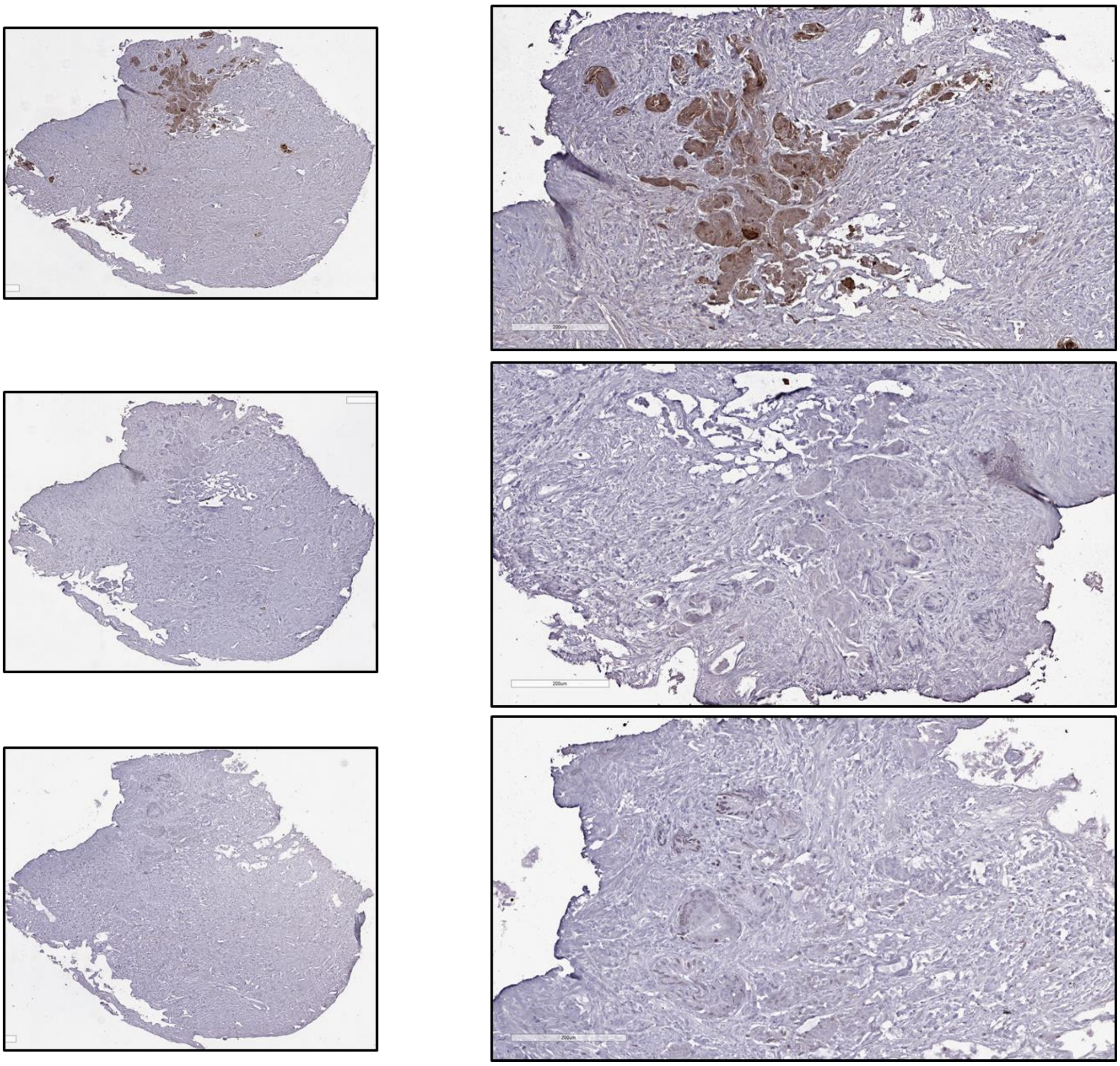

Supplementary Figure 9B. Representative images of pharmacodynamic analysis in *Ex vivo* cultures of human prostate cancer. *Ex vivo* culture of a human prostate cancer was established and treated with CYT387 (10 μM) for 48 h. Expression of AMACR (malignant marker), p63 (benign marker) and AR was assessed by IHC.

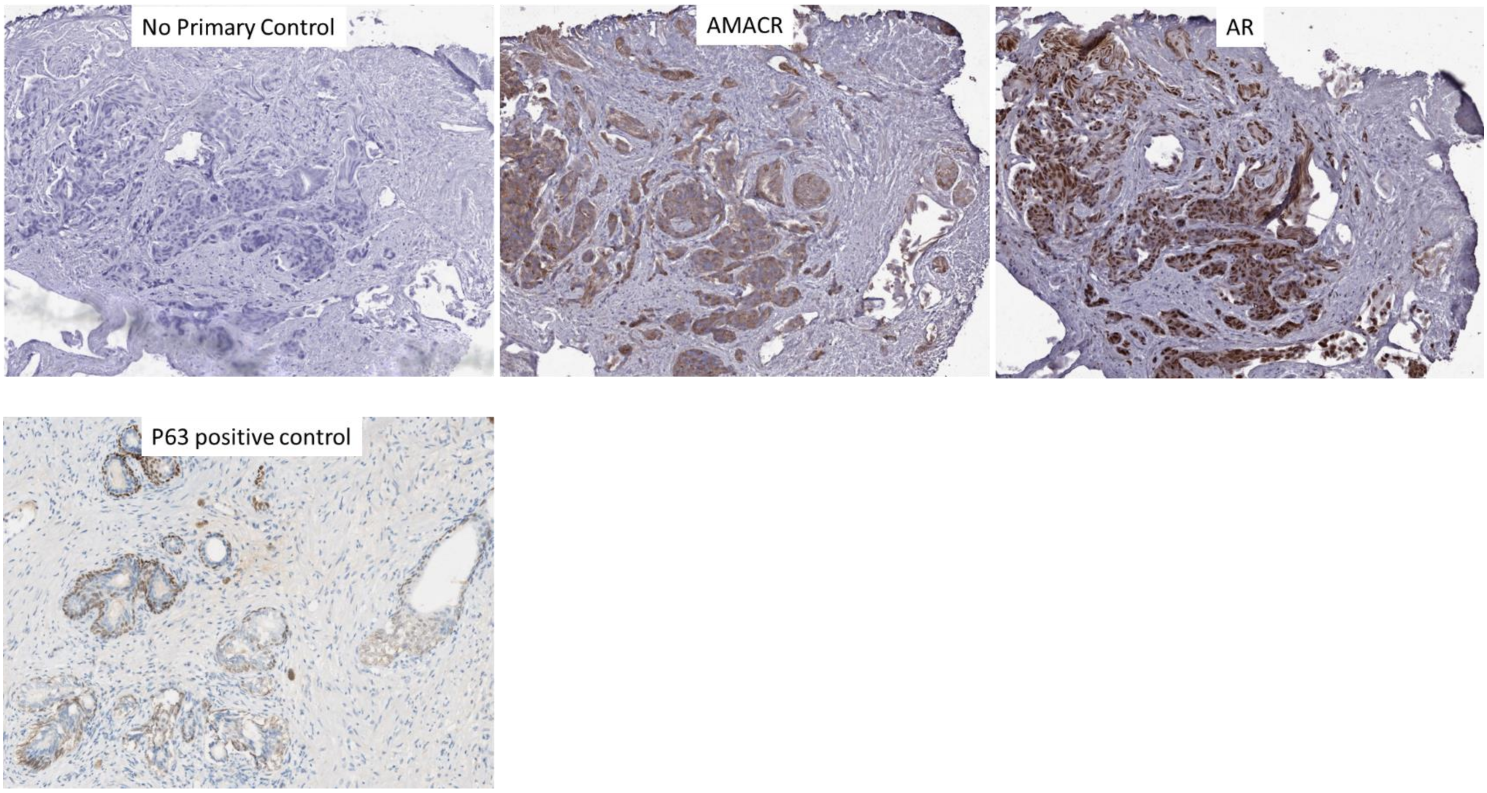

Supplementary Figure 9C. Control staining for AMACR, AR and p63.

GGTGGTactagtATGGATCCCGGGCAGCAGCCGCCGCCTCAACCGGCCCC**A**CAGGG**T**CAAGG**T**CAGCC**A**CCTT
C**A**CA**A**CC**T**CC**A**CA**A**GG**T**CA**A**GG**T**CC**T**CC**A**TC**T**GG**T**CC**A**GG**T**CAACC**A**GCACC**A**GC**A**GC**T**AC**A**CA**A**GC**A**GC**A**C
C**T**CA**A**GCACC**T**CC**A**GC**T**GG**A**CATCAGATCGTGCACGTCCGCGGGGACTCGGAGACCGACCTGGAGGCGCTCT
TCAACGCCGTCATGAACCCCAAGACGGCCAACGTGCCCCAGACCGTGCCCATGAGGCTCCGGAAGCTGCCCG
ACTCCTTCTTCAAGCCGCCGGAGCCCAAATCCCACTCCCGACAGGCCAGTACTGATGCAGGCACTGCAGGAGC
CCTGACTCCACAGCATGTTCGAGCTCAT**GCC**TCTCCAGCTTCTCTGCAGTTGGGAGCTGTTTCTCCTGGGACAC
TGACCCCCACTGGAGTAGTCTCTGGCCCAGCAGCTACACCCACAGCTCAGCATCTTCGACAGTCTTCTTTTGAG
ATACCTGATGATGTACCTCTGCCAGCAGGTTGGGAGATGGCAAAGACATCTTCTGGTCAGAGATACTTCTTAA
ATCACATCGATCAGACAACAACATGGCAGGACCCCAGGAAGGCCATGCTGTCCCAGATGAACGTCACAGCCC
CCACCAGTCCACCAGTGCAGCAGAATATGATGAACTCGGCTTCAGGTCCTCTTCCTGATGGATGGGAACAAGC
CATGACTCAGGATGGAGAAATTTACTATATAAACCATAAGAACAAGACCACCTCTTGGCTAGACCCAAGGCTT
GACCCTCGTTTTGCCATGAACCAGAGAATCAGTCAGAGTGCTCCAGTGAAACAGCCACCACCCCTGGCTCCCC
AGAGCCCACAGGGAGGCGTCATGGGTGGCAGCAACTCCAACCAGCAGCAACAGATGCGACTGCAGCAACTG
CAGATGGAGAAGGAGAGGCTGCGGCTGAAACAGCAAGAACTGCTTCGGCAGGAGTTAGCCCTGCGTAGCCA
GTTACCAACACTGGAGCAGGATGGTGGGACTCAAAATCCAGTGTCTTCTCCCGGGATGTCTCAGGAATTGAG
AACAATGACGACCAATAGCTCAGATCCTTTCCTTAACAGTGGCACCTATCACTCTCGAGATGAGAGTACAGAC
AGTGGACTAAGCATGAGCAGCTACAGTGTCCCTCGAACCCCAGATGACTTCCTGAACAGTGTGGATGAGATG
GATACAGGTGATACTATCAACCAAAGCACCCTGCCCTCACAGCAGAACCGTTTCCCAGACTACCTTGAAGCCA
TTCCTGGGACAAATGTGGACCTTGGAACACTGGAAGGAGATGGAATGAACATAGAAGGAGAGGAGCTGATG
CCAAGTCTGCAGGAAGCTTTGAGTTCTGACATCCTTAATGACATGGAGTCTGTTTTGGCTGCCACCAAGCTAG
ATAAAGAAAGCTTTCTTACATGGTTAGC**gaattcGGTGGT**

Supplementary Figure 10. Sequence of gBlock-Yap-S127A. Restriction enzyme sites are shown in blue. Transcription start site is shown in red font; codon for S127A is shown in bold red font. Bases that were codon switched are shown in bold font.

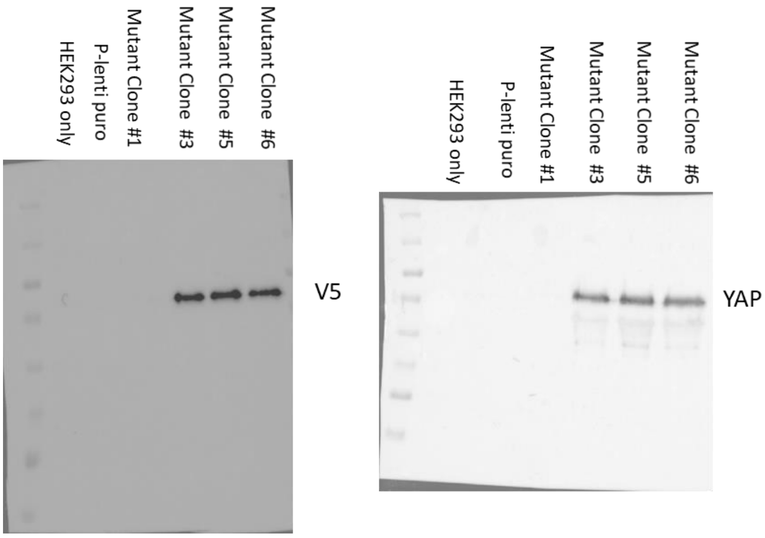

Supplementary Figure 11. Confirmation of Yap-S127A expression. HEK293T cells were transfected with pLenti-Puro-Yap S127A and protein expression confirmed using both the V5 tag antibody and the Yap antibody.

Supplementary Table 1. siRNA sequences

AR	CCAUCUUUCUGAAUGUCCU[dT][dT].
Luc	GGUACGCGGAAUACUUCGA[dT][dT]
N/S	UUCUCCGAACGUGUCACGU[dT][dT]
IKBKE 1	GCACCACAUCUAUAUCCAU[dT][dT]
IKBKE 2	ACAGAAGCAUCCAGCAGAU[dT][dT]
IKBKE 3	GAAAGGACCUGCUUCUCCA[dT][dT]
Myc 1	CGUCCAAGCAGAGGAGCAA[dT][dT]
Myc 2	GCUUGUACCUGCAGGAUCU[dT][dT]
Yap 1	CACCUAUCACUCUCGAGAU[dT][dT]
Yap 2	GCUCAUUCCUCUCCAGCUU[dT][dT]

Supplementary Table 2. qPCR primers

AR Forward	GTGCAGCCTATTGCGAGAG
AR Reverse	ATCTGAAAGGGGGCATGAGC
CCNA2 Forward	GAAGACGAGACGGGTTGCA
CCNA2 Reverse	AGGAGGAACGGTGACATGCT
FKBP5 Forward	GAATACACCAAAGCTGTTGA
FKBP5 Reverse	CTCTTCCTTGGCATCCT
HPRT1 Forward	TTGCTTTCCTTGGTCAGGCA
HPRT1 Reverse	AGCTTGCGACCTTGACCATCT
IKBKE Forward	TGACAGTGAGCCCTGAAAGC
IKBKE Reverse	CCGGATTTCCCACACTCTGA
MYC Forward	GTAGTGGAAAACCAGCAGC
MYC Reverse	GAAGCTAACGTTGAGGGGCA
PSA Forward	TCGGCACAGCCTGTTTCAT
PSA Reverse	TGGCTGACCTGAAATACCTGG
TBK1 Forward	CACATTTTAGAAAGGTTTGGCTC
TBK1 Reverse	CTATCTATAAAGGCATTCATTTAATGC
TMPRSS2 Forward	TGTCTCCCTGCACCACTAACTAG
TMPRSS2 Reverse	GCAAACATTGAAAAGAGCCTC
AR-V7 Forward	AAGAGAAGTACCTGTGCGCC
AR-V7 Reverse	TCAGGGTCTGGTCATTTTGA
YAP Forward	TGCGTAGCCAGTTACCAACACTG
YAP Reverse	TCGAGAGTGATAGGTGCCACTG

Supplementary Table 3. ChIP primers

Myc binding site within the AR gene (Forward) (21)	ACTGAGGAGACAACCCAG
Myc binding site within the AR gene (Reverse) (21)	TCTCATGCTCCCACTTCC
AREI (Forward)	CCTAGATGAAGTCTCCATGAGCTACA
AREI (Reverse)	GGGAGGGAGAGCTAGCACTTG
AREII (Forward) (22)	AGGGATCAGGGAGTCTCACA
AREII (Reverse) (22)	GCTAGCACTTGCTGTTCTGC
AREIII (Forward)	TGGGACAACTTGCAAACCTG
AREIII (Reverse)	CCAGAGTAGGTCTGTTTTCAATCCA

Supplementary Table 4. Ex vivo culture patient information.

**Patient sample**	**Age**	**Pathological stage**	**Gleason Grade**	**PSA at diagnosis (ng/ml)**	**Prior treatments**
14433 (chTURP)	73	T4 N1 M0	5+4=9	12.9	LHRH therapy
REX24 (RRP)	69	T3 N0 M0	3+4=7	3.6	None
14436 (chTURP)	74		4+5=9	N/A	Radiotherapy

Supplementary References

1. Rigas, A.C., Ozanne, D.M., Neal, D.E. and Robson, C.N. (2003) The scaffolding protein RACK1 interacts with androgen receptor and promotes cross-talk through a protein kinase C signaling pathway. *J Biol Chem*, **278**, 46087-46093.
2. Walker, S., Foster, F., Wood, A., Owens, T., Brennan, K., Streuli, C.H. and Gilmore, A.P. (2016) Oncogenic activation of FAK drives apoptosis suppression in a 3D-culture model of breast cancer initiation. *Oncotarget*, **7**, 70336-70352.
3. Schindelin, J., Arganda-Carreras, I., Frise, E., Kaynig, V., Longair, M., Pietzsch, T., Preibisch, S., Rueden, C., Saalfeld, S., Schmid, B. *et al.* (2012) Fiji: an open-source platform for biological-image analysis. *Nat Methods*, **9**, 676-682.
4. Cerami, E., Gao, J., Dogrusoz, U., Gross, B.E., Sumer, S.O., Aksoy, B.A., Jacobsen, A., Byrne, C.J., Heuer, M.L., Larsson, E. *et al.* (2012) The cBio cancer genomics portal: an open platform for exploring multidimensional cancer genomics data. *Cancer Discov*, **2**, 401-404.
5. Gao, J., Aksoy, B.A., Dogrusoz, U., Dresdner, G., Gross, B., Sumer, S.O., Sun, Y., Jacobsen, A., Sinha, R., Larsson, E. *et al.* (2013) Integrative analysis of complex cancer genomics and clinical profiles using the cBioPortal. *Sci Signal*, **6**, pl1.
6. Taylor, B.S., Schultz, N., Hieronymus, H., Gopalan, A., Xiao, Y., Carver, B.S., Arora, V.K., Kaushik, P., Cerami, E., Reva, B. *et al.* (2010) Integrative genomic profiling of human prostate cancer. *Cancer Cell*, **18**, 11-22.
7. Barbieri, C.E., Baca, S.C., Lawrence, M.S., Demichelis, F., Blattner, M., Theurillat, J.P., White, T.A., Stojanov, P., Van Allen, E., Stransky, N. *et al.* (2012) Exome sequencing identifies recurrent SPOP, FOXA1 and MED12 mutations in prostate cancer. *Nat Genet*, **44**, 685-689.
8. Grasso, C.S., Wu, Y.M., Robinson, D.R., Cao, X., Dhanasekaran, S.M., Khan, A.P., Quist, M.J., Jing, X., Lonigro, R.J., Brenner, J.C. *et al.* (2012) The mutational landscape of lethal castration-resistant prostate cancer. *Nature*, **487**, 239-243.
9. Baca, S.C., Prandi, D., Lawrence, M.S., Mosquera, J.M., Romanel, A., Drier, Y., Park, K., Kitabayashi, N., MacDonald, T.Y., Ghandi, M. *et al.* (2013) Punctuated evolution of prostate cancer genomes. *Cell*, **153**, 666-677.
10. Hieronymus, H., Schultz, N., Gopalan, A., Carver, B.S., Chang, M.T., Xiao, Y., Heguy, A., Huberman, K., Bernstein, M., Assel, M. *et al.* (2014) Copy number alteration burden predicts prostate cancer relapse. *Proc Natl Acad Sci U S A*, **111**, 11139-11144.
11. Cancer Genome Atlas Research, N. (2015) The Molecular Taxonomy of Primary Prostate Cancer. *Cell*, **163**, 1011-1025.
12. Robinson, D., Van Allen, E.M., Wu, Y.M., Schultz, N., Lonigro, R.J., Mosquera, J.M., Montgomery, B., Taplin, M.E., Pritchard, C.C., Attard, G. *et al.* (2015) Integrative Clinical Genomics of Advanced Prostate Cancer. *Cell*, **162**, 454.
13. Beltran, H., Prandi, D., Mosquera, J.M., Benelli, M., Puca, L., Cyrta, J., Marotz, C., Giannopoulou, E., Chakravarthi, B.V., Varambally, S. *et al.* (2016) Divergent clonal evolution of castration-resistant neuroendocrine prostate cancer. *Nat Med*, **22**, 298-305.
14. Kumar, A., Coleman, I., Morrissey, C., Zhang, X., True, L.D., Gulati, R., Etzioni, R., Bolouri, H., Montgomery, B., White, T. *et al.* (2016) Substantial interindividual and limited intraindividual genomic diversity among tumors from men with metastatic prostate cancer. *Nat Med*, **22**, 369-378.
15. Abida, W., Armenia, J., Gopalan, A., Brennan, R., Walsh, M., Barron, D., Danila, D., Rathkopf, D., Morris, M., Slovin, S. *et al.* (2017) Prospective Genomic Profiling of Prostate Cancer Across Disease States Reveals Germline and Somatic Alterations That May Affect Clinical Decision Making. *JCO Precis Oncol*, **2017**.

16. Fraser, M., Sabelnykova, V.Y., Yamaguchi, T.N., Heisler, L.E., Livingstone, J., Huang, V., Shiah, Y.J., Yousif, F., Lin, X., Masella, A.P. *et al.* (2017) Genomic hallmarks of localized, non-indolent prostate cancer. *Nature*, **541**, 359-364.
17. Armenia, J., Wankowicz, S.A.M., Liu, D., Gao, J., Kundra, R., Reznik, E., Chatila, W.K., Chakravarty, D., Han, G.C., Coleman, I. *et al.* (2018) The long tail of oncogenic drivers in prostate cancer. *Nat Genet*, **50**, 645-651.
18. Gerhauser, C., Favero, F., Risch, T., Simon, R., Feuerbach, L., Assenov, Y., Heckmann, D., Sidiropoulos, N., Waszak, S.M., Hubschmann, D. *et al.* (2018) Molecular Evolution of Early-Onset Prostate Cancer Identifies Molecular Risk Markers and Clinical Trajectories. *Cancer Cell*, **34**, 996-1011 e1018.
19. Ren, S., Wei, G.H., Liu, D., Wang, L., Hou, Y., Zhu, S., Peng, L., Zhang, Q., Cheng, Y., Su, H. *et al.* (2018) Whole-genome and Transcriptome Sequencing of Prostate Cancer Identify New Genetic Alterations Driving Disease Progression. *Eur Urol*, **73**, 322-339.
20. Abida, W., Cyrta, J., Heller, G., Prandi, D., Armenia, J., Coleman, I., Cieslik, M., Benelli, M., Robinson, D., Van Allen, E.M. *et al.* (2019) Genomic correlates of clinical outcome in advanced prostate cancer. *Proc Natl Acad Sci U S A*, **116**, 11428-11436.
21. Lee, J.G., Zheng, R., McCafferty-Cepero, J.M., Burnstein, K.L., Nanus, D.M. and Shen, R. (2009) Endothelin-1 enhances the expression of the androgen receptor via activation of the c-myc pathway in prostate cancer cells. *Mol Carcinog*, **48**, 141-149.
22. Shang, Y., Myers, M. and Brown, M. (2002) Formation of the androgen receptor transcription complex. *Mol Cell*, **9**, 601-610.
